# Modeling and Rescue of RP2 Retinitis Pigmentosa Using iPSC-Derived Retinal Organoids

**DOI:** 10.1016/j.stemcr.2020.05.007

**Published:** 2020-06-11

**Authors:** Amelia Lane, Katarina Jovanovic, Ciara Shortall, Daniele Ottaviani, Anna Brugulat Panes, Nele Schwarz, Rosellina Guarascio, Matthew J. Hayes, Arpad Palfi, Naomi Chadderton, G. Jane Farrar, Alison J. Hardcastle, Michael E. Cheetham

**Affiliations:** 1 UCL Institute of Ophthalmology, London, UK; 2 Smurfit Institute of Genetics, Trinity College Dublin, Dublin 2, Ireland

**Keywords:** retinal degeneration, retinal organoid, gene therapy, CRISPR gene editing, genetic, inherited disease, disease modeling, stem cell, AAV, cell death

## Abstract

*RP2* mutations cause a severe form of X-linked retinitis pigmentosa (XLRP). The mechanism of *RP2*-associated retinal degeneration in humans is unclear, and animal models of *RP2* XLRP do not recapitulate this severe phenotype. Here, we developed gene-edited isogenic *RP2* knockout (RP2 KO) induced pluripotent stem cells (iPSCs) and *RP2* patient-derived iPSC to produce 3D retinal organoids as a human retinal disease model. Strikingly, the RP2 KO and *RP2* patient-derived organoids showed a peak in rod photoreceptor cell death at day 150 (D150) with subsequent thinning of the organoid outer nuclear layer (ONL) by D180 of culture. Adeno-associated virus-mediated gene augmentation with human RP2 rescued the degeneration phenotype of the RP2 KO organoids, to prevent ONL thinning and restore rhodopsin expression. Notably, these data show that 3D retinal organoids can be used to model photoreceptor degeneration and test potential therapies to prevent photoreceptor cell death.

## Highlights

• Isogenic RP2 knockouts match the phenotype of RP2 patient-derived organoids
• Retinal organoids from RP2 nulls undergo rod photoreceptor cell death
• Cell death correlates with rod photoreceptor differentiation
• AAV gene therapy improved photoreceptor viability and increased rhodopsin expression

## Graphical Abstract

**Figure:**
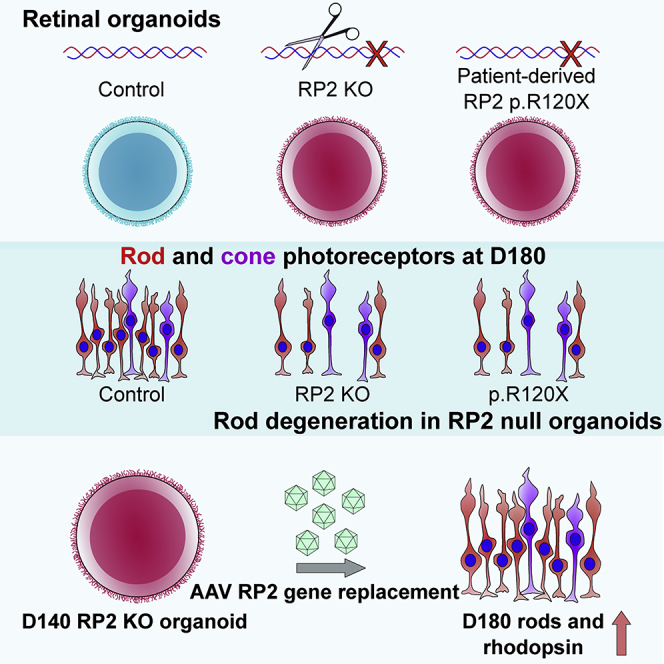

## Teaser

Cheetham and colleagues show that 3D retinal organoids lacking the RP2 protein develop rod photoreceptor degeneration that can be prevented with AAV gene augmentation for RP2.

## Introduction

The reprogramming of patient-derived cells into induced pluripotent stem cells (iPSCs) has enabled the derivation and differentiation of a range of somatic cell types and has revolutionized our ability to study inherited disease (Takahashi et al., 2007). The differentiation of iPSCs toward retinal lineages has seen huge advances in recent years with the refinement of protocols for the generation of 3D retinal organoids (ROs) (Gagliardi et al., 2019, Nakano et al., 2012). Unlike previous models in 2D, these 3D structures contain photoreceptors with morphologically identifiable features; including, inner segments rich in mitochondria, rudimentary outer segments with connecting cilia, and synaptic pedicles, in addition to bipolar, Müller glia, ganglion and amacrine cells, synaptic layers, and an outer limiting membrane (OLM), arranged in retinal layers (reviewed in Capowski et al., 2019). These advanced models have proven to have many translational research applications, including transplantation studies (Gonzalez-Cordero et al., 2017, Shirai et al., 2016), retinal disease modeling, and testing the efficacy of potential therapies in human photoreceptor cells (Deng et al., 2018, Parfitt et al., 2016, Schwarz et al., 2017, Sharma et al., 2017). To date, however, they have not been used to model and rescue photoreceptor cell death.

Mutations in *RP2* account for approximately 15% of all cases of X-linked retinitis pigmentosa (XLRP) (Breuer et al., 2002, Hardcastle et al., 1999). RP2 is a GTPase-activating protein (GAP) for the small GTPase ARL3 (Veltel et al., 2008), which is also regulated by its guanine nucleotide exchange factor (GEF) ARL13B (Gotthardt et al., 2015). ARL13B is localized to the ciliary axoneme, whereas a pool of RP2 and ARL3 localize at the basal body and associated centriole at the base of photoreceptors (Evans et al., 2010, Grayson et al., 2002). ARL3, with its effectors (UNC119 and PDEdelta [PDED]) and GAP RP2, are thought to be important in the retina to traffic lipidated proteins, such as transducin, GRK1, and PDE6, to the photoreceptor outer segment (Ismail et al., 2011, Schwarz et al., 2012, Wright et al., 2011, Zhang et al., 2011, Zhang et al., 2015). *Rp2* knockout mice have a relatively mild phenotype compared with human disease. In one model mis-localization/absence of GRK1 and cone PDE6a was evident at 14 months (Zhang et al., 2015), whereas another model was reported to have rhodopsin and M opsin mis-localization at 2 months and outer nuclear layer (ONL) thinning at 5 months (Li et al., 2013). In contrast, the human phenotype is relatively severe with some patients experiencing macular atrophy in childhood (Jayasundera et al., 2010), highlighting the necessity for human retinal models of disease.

Currently there are no treatments for this condition, so there is a need to develop potential therapies. Characterization of iPSC-derived RPE and early-stage ROs from an individual carrying a nonsense mutation in *RP2* (c.358C > T, p.R120X) showed changes in Golgi cohesion, Gbeta trafficking in RPE, and ciliary trafficking of KIF7 in ROs (Schwarz et al., 2015, Schwarz et al., 2017). Furthermore, treatment with the readthrough drugs, G418 and/or Ataluren (PTC124), could restore detectable full-length RP2 protein and rescue the Golgi cohesion and Gbeta mis-localization in iPSC-RPE and kinesin traffic in ROs. Gene therapy for other inherited retinal diseases using adeno-associated viruses (AAVs) has been shown to efficiently transduce photoreceptors and RPE following subretinal injection (Sarra et al., 2002) in animal models. There is a Food and Drug Administration- and European Medicines Agency-approved AAV-mediated ocular gene therapy (Russell et al., 2017), and a number of AAV-mediated ocular gene therapies are currently in phase I/II and III clinical trials (clinicaltrials.gov). AAV delivery of human RP2 to a mouse knockout model of RP2-XLRP preserved cone function, but had no effect on rod cell function and toxicity was observed at a higher viral dose (Mookherjee et al., 2015).

Here, we describe the temporal maturation of CRISPR gene-edited *RP2* knockout ROs relative to their isogenic control, in addition to ROs derived from two unrelated individuals with the same R120X nonsense mutation. These studies reveal that the loss of RP2 leads to rod photoreceptor degeneration that can be rescued by AAV delivery of RP2.

## Results

### *RP2* Knockout and *RP2* Patient iPSCs Develop Mature ROs

**Figure 1:**
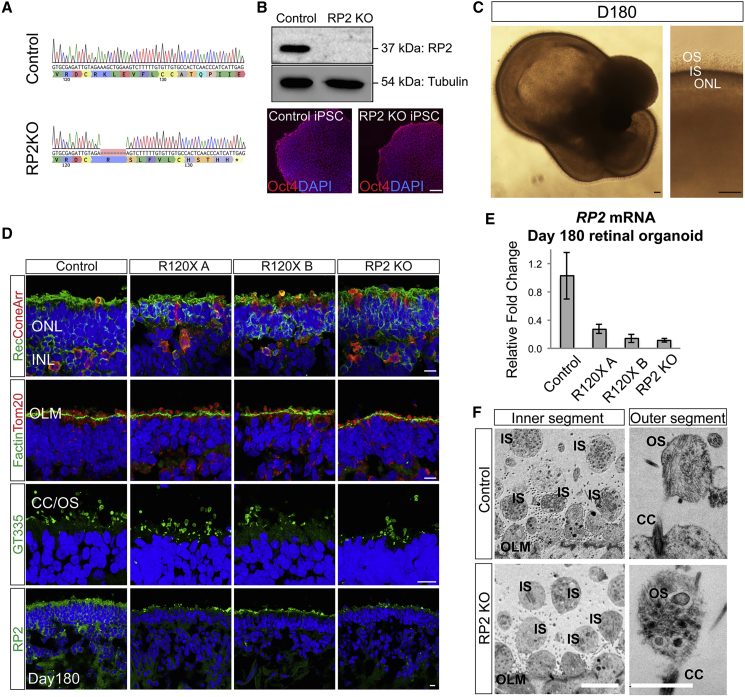
Retinal Organoids from R120X, RP2 KO, and Isogenic Control iPSCs (A) Sanger sequence trace of edited RP2 KO iPSCs. CRISPR/Cas9 gene editing was used to create an 8-bp deletion in exon 2 of *RP2* by NHEJ. (B) Western blot and immunocytochemistry (ICC) of control and RP2 KO iPSC. Scale bar, 50 μm. (C) Retinal organoid morphology at D180, a layer of IS/OS is visible by differential interference contrast above the transparent ONL. Scale bar, 50 μm. (D) ICC of retinal organoids at D180. Photoreceptors (recoverin and cone arrestin [coneArr]), outer limiting membrane (OLM, F actin) mitochondria (Tom20), and connecting cilia/OS (GT335) expression in the ONL of control and RP2 null iPSC retinal organoids. RP2 is expressed at the plasma membrane of cells in the ONL and inner nuclear layer (INL) in control organoids. Scale bar, 10 μm. (E) qPCR of *RP2* mRNA in whole retinal organoids at D180 (n = 3 independent organoids). Mean ± standard error of the mean (SEM). (F) Electron micrographs of control and RP2 KO retinal organoids at D180. Scale bars, 5 μm (left), 1 μm (right).

Fibroblasts from two unrelated individuals (R120X-A and R120X-B) carrying the nonsense mutation c.358C > T; p.R120X were reprogrammed into iPSCs by nucleofection (Okita et al., 2011, Schwarz et al., 2015). CRISPR/Cas9 with guides designed to target exon 2 of *RP2* were used to generate RP2 knockout iPSCs using a simultaneous reprogramming and gene-editing protocol (Howden et al., 2015). The location of the gRNA on exon 2 was selected due to its proximity to the c.358C > T p.R120X site, thus any knockout lines generated would closely mimic the consequences of this nonsense mutation. RP2 KO iPSC clones were identified through non-homologous end-joining-mediated creation of indels; one clone with an 8-bp deletion in exon 2 of *RP2* (RP2 c.371_378delAAGCTGGA; p.Lys124SerfsTer11) was selected for further study as it had the shortest frameshift extension before a premature stop codon. Western blotting of iPSCs showed efficient knockout, as no RP2 protein was detectable (Figures 1A and 1B).

The RP2 KO line, the non-edited isogenic control, and two R120X patient iPSC lines were differentiated into ROs over a period of 4–10 months using methods described previously (Nakano et al., 2012, Zhong et al., 2014) with slight modifications. Both methods produced biologically similar organoids that were developmentally and structurally comparable at the time points tested. By day 180 (D180) a transparent ONL with a brush-like border was visible by light microscopy (Figure 1C). Scattered rhodopsin-positive cells were first detectable in control organoids in the recoverin-positive ONL from D150 then increased in number over time as the ROs matured up to D180 (Figure S1A). By D180 all cell lines were able to generate ROs consisting of a laminated structure with a compacted ONL containing recoverin and cone arrestin-positive photoreceptors (Figure 1D), above an inner nuclear layer (INL) containing protein kinase C alpha (PKCα)-positive bipolar cells and CRALBP/Nestin-positive Müller glia (Figure S1B). In all ROs, the ONL terminated at the apical edge, with an OLM that was strongly immunoreactive for F actin (Figure 1D). Above the OLM, mitochondria (immunoreactive for TOM20) were enriched in globular inner segments. Immunostaining for the ciliary marker ARL13B and polyglutamylated tubulin (GT335) revealed the bulging shape at the tip of the photoreceptor connecting cilia, as they matured to form outer segment (OS) like structures from D150 onward (Figure S1C). RP2 could be detected in control ROs at the plasma membrane in all cells in the INL and ONL. In RP2 KO and R120X cell lines, RP2 immunoreactivity was absent by immunocytochemistry (ICC), although some background fluorescence was observed at the edges of the presumptive OS. qPCR was used to measure relative *RP2* mRNA expression in ROs at D180. The RP2 KO clone had only 20% *RP2* mRNA relative to its isogenic control, similar to the R120X patient ROs (n = 3) (Figure 1E), suggesting that the mutant allele transcript is subject to nonsense-mediated decay in all these ROs. Electron microscopy confirmed the presence of membranous-rich structures at the apical ciliary tip, reminiscent of early OS formation, in both controland RP2 KO ROs (Figure 1F). These rudimentary OS were often found detached from the body of the RO indicating the flexibility, or fragility, of these structures in the absence of RPE. Collectively, these results show that RP2 ablation does not prevent the differentiation of iPSC into photoreceptors bearing OS-like structures in 3D RO culture.

### Loss of RP2 Leads to Photoreceptor Cell Death and ONL Thinning

**Figure 2:**
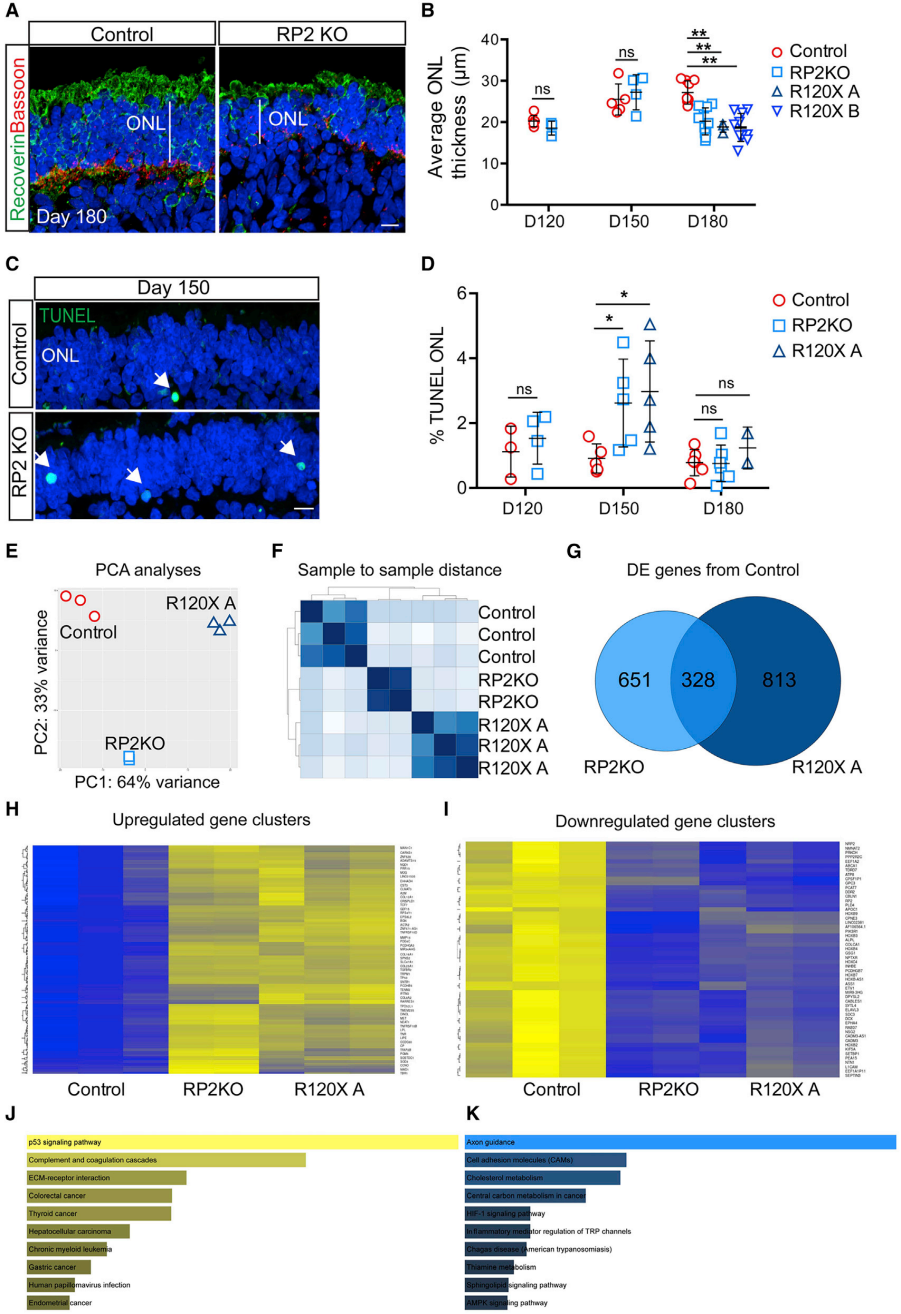
Photoreceptor Differentiation-Associated Cell Death in RP2 KO Organoids (A) ICC of control and RP2 KO retinal organoids showing reduced ONL thickness in RP2 KO. Recoverin staining demarcates the ONL terminating in the synaptic layer stained with Bassoon. Scale bar, 10 μm. (B) Mean ONL thickness per organoid was measured from tilescans of cryosections of a whole organoid at D120, D150 (n = 5 control; n = 4 RP2 KO at both time points), and D180. Significant ONL thinning was recorded at D180 in RP2 KO (n = 9 independent organoids) and R120X lines (n = 3 R120X A organoids, n = 9 R120X B organoids), but not in controls (n = 10 independent organoids; p ≤ 0.01; mean ± SD). (C) TUNEL reactive nuclei (arrows) in the ONL of RP2 KO and isogenic control organoids at D150. Scale bar, 10 μm. (D) Quantification of TUNEL reactivity. RP2 KO organoids had a significantly higher proportion of TUNEL-positive cells at D150 (n = 5 independent organoids p ≤ 0.05, mean ± SD) but not at D120 (n = 3 control; n = 4 RP2 KO) or D180 (n = 5 control; n = 6 RP2 KO). R120X organoids also had increased TUNEL reactivity at D150 (n = 5 at D150 n = 2 at D180 independent organoids). (E) Principal-component analyses of RNA-seq data from ROs (n = 3 control and R120X A, n = 2 RP2 KO independent organoids). (F) Sample to sample distance between samples. (G) Venn diagram showing differentially expressed genes between RP2 KO and control and R120X and control and common genes. (H) Heatmap showing upregulated clusters of differentially expressed genes (blue, lower expression; yellow, higher expression). (I) Heatmap of downregulated clusters of differentially expressed genes. (J) KEGG pathway analyses of upregulated genes. (K) KEGG pathway analyses of downregulated genes.

Similar to the neural retina *in vivo*, the outermost cell layer of the ROs consists of a uniform compacted ONL, which terminates with photoreceptor synaptic pedicles that are separated from the inner retinal cells by a layer immunoreactive for synaptic structural protein Bassoon in the outer plexiform layer (Figure 2A). A reduction in the number of photoreceptor nuclei in the ONL, and thereby thickness, *in vivo* is a marker of photoreceptor cell degeneration. Therefore, ONL thickness was measured in isogenic controls and RP2 KO at D120, D150, and D180 (Figure 2B). In control ROs, the average ONL thickness increased between D120 and D150 from 20 to 25 μm, then did not change significantly between D150 and D180. In contrast, in RP2 KO ROs the average ONL thickness decreased significantly between D150 and D180 (p = 0.02). Similarly, R120X ROs from both patients had significantly thinner ONLs at D180 compared with the control cell line (p ≤ 0.01; Figures 2 and S2). This suggested that photoreceptor cell death might be occurring between D150 and D180 in the RP2 null cell lines.

To test this hypothesis further, we measured TUNEL reactivity across the ONL in RP2 KO and control ROs at D120, D150, and D180 (Figures 2C and 2D). A small percentage of TUNEL-positive nuclei were detectable in the photoreceptor ONL at all time points. In control ROs, TUNEL reactivity was not significantly different at D120, D150, or D180. Whereas, the RP2 KO ROs had a significantly higher percentage of TUNEL-positive cells in the ONL at D150. There was no significant difference between RP2 KO and controls at D120 or D180, suggesting a peak of cell death during photoreceptor differentiation and maturation around D150 in RP2-deficient cell lines. This was confirmed in R120X-A ROs, with an increase in TUNEL reactivity at D150, which had resolved by D180 (Figure 2D). These data show that there is a peak in photoreceptor cell death that correlates with their maturation and the time course of increased rhodopsin expression.

### Gene Expression Changes Associated with Loss of RP2

To investigate gene expression changes that might be associated with the death of photoreceptors in the RP2 null organoids, RNA sequencing (RNA-seq) was performed on D150 controls, RP2 KO, and R120X-A ROs. Principal-component analyses and sample to sample distance shows that the gene expression profiles of the RP2 KO ROs were between the R120X-A patient line and the parental isogenic control (Figures 2E and 2F). There were 328 shared differentially expressed (DE) genes between the RP2 KO and R120X ROs compared with control (Figures 2G–2I). By contrast there were 651 and 813 DE genes between control and RP2 KO and R120X-A ROs, respectively, that were not shared. Kyoto Encyclopedia of Genes and Genomes (KEGG) pathway analyses of the shared DE genes revealed that the “p53 signaling pathway” was the major upregulated pathway, whereas the major downregulated pathway was “axon guidance” (Figures 2J and 2K). KEGG pathway analyses of the axon guidance-related changes showed that expression of genes in pathways that stimulate axon outgrowth, attraction, and repulsion were reduced (Figure S3). Whereas further investigation of the KEGG apoptosis and p53 signaling pathways showed that a number of pro-apoptotic genes, such as p21, BAX, and PUMA, were upregulated in both RP2 null ROs (Figure S3), supporting the observation that loss of RP2 induces cell death in ROs.

### Rod Photoreceptor Differentiation and Survival Is Compromised in RP2 KO and R120X ROs

**Figure 3:**
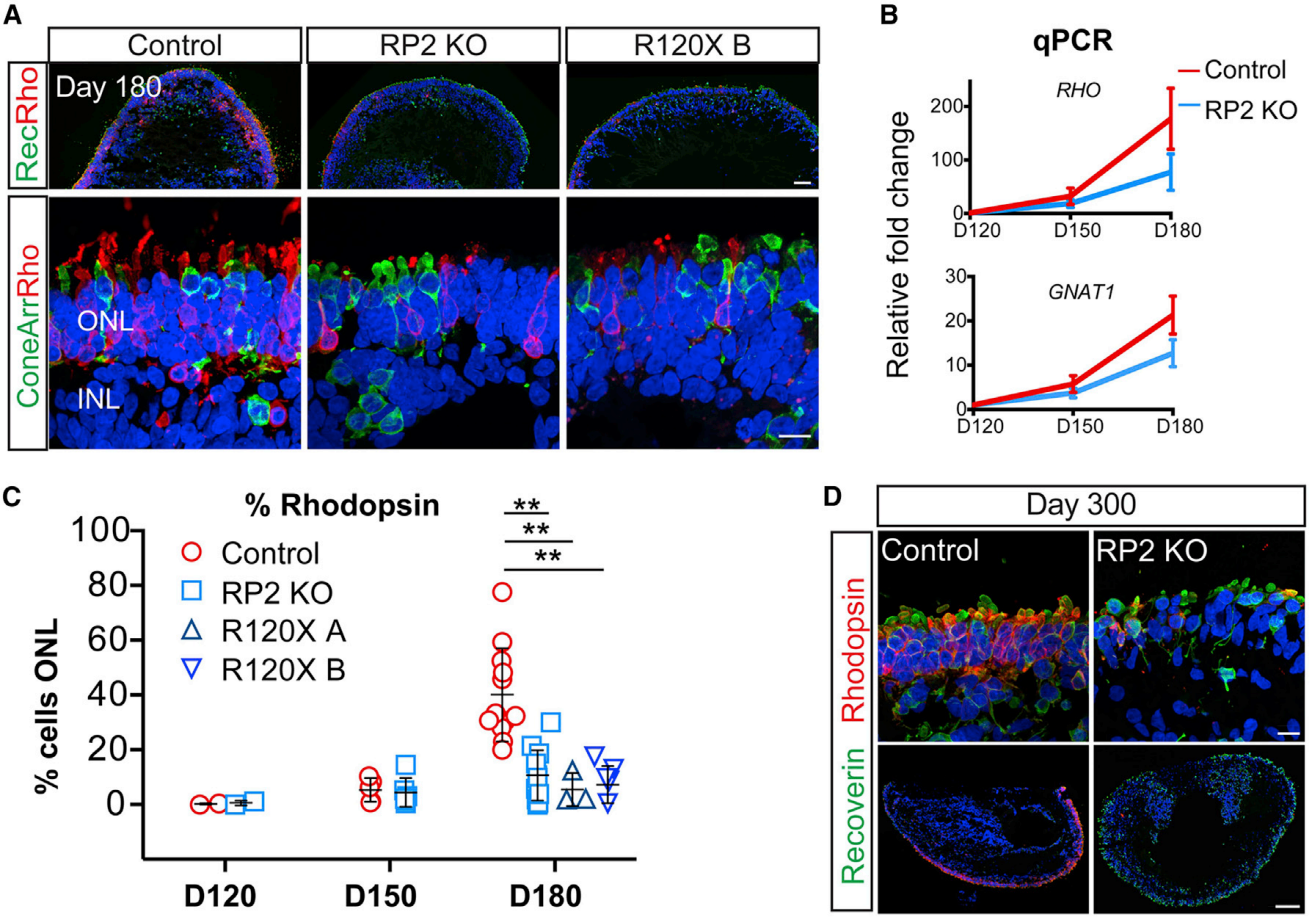
Reduced Number of Rod Cells in RP2 KO and R120X Patient Retinal Organoids (A) ICC of retinal organoids. Low (upper panel) and high (lower panel) power magnification of recoverin, rhodopsin, and cone arrestin immunoreactivity in the ONL at D180 in control, RP2 KO, and R120X RP2 retinal organoids. Scale bars, 50 μm and 10 μm. (B) *RHO* and *GNAT1* levels in retinal organoids. qPCR showing relative fold change in mRNA in control and RP2 KO retinal organoids at D120, D150, and D180 (n = 3, 3, 4 independent organoids). Mean ± SEM. (C) Quantification of rhodopsin-positive cells as a percentage of ONL in control, RP2 KO and R120X patient cell lines from D120 to D180 of differentiation. Each data point represents the mean of 1 independent organoid, counts are from tilescans of whole organoid cross-sections (D120 n = 2 control, n = 2 RP2 KO; D150 n = 5 control, n = 6 RP2 KO; D180 n = 12 control, n = 11 RP2 KO, n = 3 R120X A, n = 5 R120X B; ^∗∗^p ≤ 0.01; mean ± SD). (D) High and low magnification of control and RP2 KO organoids at D300 of differentiation stained with recoverin and rhodopsin. Scale bars, 10 μm (upper panel) and 100 μm (lower panel).

To identify which types of photoreceptor cells were most affected by the loss of RP2, rods and cones were stained with rhodopsin and cone arrestin at D180 (Figure 3A). Strikingly, in the RP2 KO ROs, there was reduced immunoreactivity for rhodopsin compared with isogenic controls. Unlike recoverin expression, which was widespread in the RP2 KO ROs, rhodopsin was restricted to patches of the ONL. To assess photoreceptor gene expression, we quantified rhodopsin (*RHO*) and rod transducin (*GNAT1*) expression by qPCR. In the controls both *RHO* and *GNAT1* showed an age-dependent increase, with a major increase between D150 and D180. In contrast, the RP2 KO ROs had reduced expression of *RHO* and *GNAT1* mRNA at D180, but not D120 or D150, confirming that this difference manifested from D150 onward. The percentage of photoreceptors that were immunoreactive for rhodopsin was quantified as a percentage of DAPI-positive nuclei across the full length of the ONL in sections from all ROs (Figure 3C). The controls showed an increase in rhodopsin-positive cells as the ROs matured. Despite variation between individual ROs, there was a significant difference in the percentage of rhodopsin-expressing cells in mature ROs (D180) between the isogenic control and RP2 KO cell lines (Figures 3A and 3C). Furthermore, the two R120X lines also showed very few rhodopsin-positive photoreceptors at D180, similar to the RP2 KO ROs (Figure 3C). By contrast, the percentage of cone arrestin-positive cells was significantly increased in the RP2 KO and R120X ROs compared with controls at D180, and the mRNA (*ARR3*) was also increased in the RP2 KO ROs at D150 and D180 (Figure S4), suggesting that the defect primarily affects rods and not cones. There was no significant difference in bipolar cell numbers (Chx10/PKCα) between control and RP2 KO ROs (Figure S4). To exclude that these differences might be attributed to simply a delay in the rate of maturation between the cell lines, control and RP2 KO ROs were maintained in culture for a further 120 days to D300. The RP2 KO had fewer rhodopsin-positive cells relative to the control ROs at D300, whereas recoverin-positive photoreceptors were maintained (Figure 3E).

### AAV2/5 Efficiently Transduces ROs to Augment RP2 Expression in RP2 Null Photoreceptors

**Figure 4:**
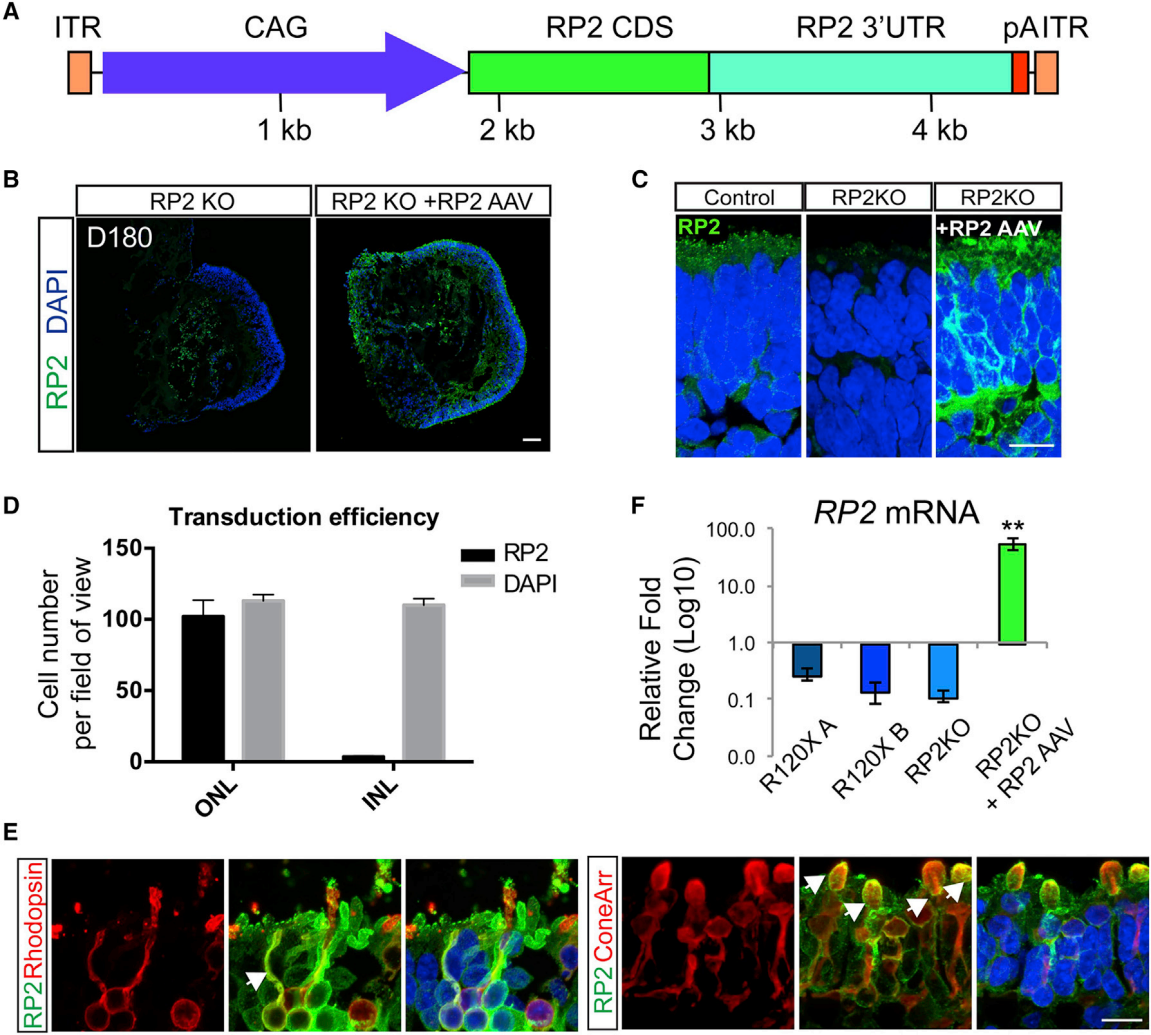
AAV2/5 RP2 Efficiently Transduces Rod and Cone Photoreceptors (A) Schematic of AAV construction. CAG promoter (blue); RP2 CDS (green); RP2 3′ UTR (teal); pA (red, minimal rabbit b globin poly A); ITR (orange). (B) RP2 expression in RP2 KO retinal organoids after transduction. ICC on RP2 KO retinal organoid cryosections 6 weeks after transduction with AAV2/5 CAG RP2 showing RP2 expression across the photoreceptor layer (ONL). Scale bar, 50 μm. (C) High-power magnification of RP2 immunoreactivity. Scale bar, 10 μm. (D) Cells with RP2 immunoreactivity in the ONL and INL scored against DAPI (mean = 90% ± 7% ONL versus 3% ± 0.4% INL, n = 3 independent organoids; mean ± SD). (E) ICC co-staining RP2 with cone arrestin or rhodopsin showing AAV-driven RP2 expression in both rod and cone photoreceptors. Scale bar, 10 μm. (F) qPCR of RP2 mRNA transcript levels in AAV-transduced RP2 KO organoids relative to endogenous expression in control organoids (n = 3 independent organoids; mean = 55-fold ±7.7 SEM).

AAVs are able to transduce post-mitotic rods and cones in mice following subretinal injection (Sarra et al., 2002) and in iPSC-derived rod and cone photoreceptors in ROs with varying efficiency (Gonzalez-Cordero et al., 2018). To assess the ability of AAVs to deliver RP2 to deficient photoreceptors, RP2 KO ROs were transduced with AAV2/5.CAGp.RP2 (Figure 4A) at D140, before the observed onset of ONL thinning, and harvested at D180. At D180, RP2 protein could be detected by ICC throughout the ONL (Figure 4B). At higher magnification, the RP2 signal was detected on the plasma membrane of photoreceptors (Figure 4C). The percentage of RP2-positive cells was determined by scoring nuclei with closely adjacent RP2 signal on the plasma membrane that matched that cell morphology. The transduction efficiency was 90% ± 7% in the ONL (Figure 4D). This included both rhodopsin-positive rod cells (Figure 4E) and cone arrestin-positive cone cells (Figure 4E). Sporadic RP2 staining in the inner retinal layers was also visible, suggesting that a small proportion of the AAV is able to penetrate the RO and transduce inner retinal cells (Figure S5). Analysis of mRNA levels by qPCR revealed an average 55-fold increase in RP2 transcript relative to control organoids (Figure 4F).

### AAV2/5-Driven RP2 Gene Augmentation Improves Photoreceptor Survival

**Figure 5:**
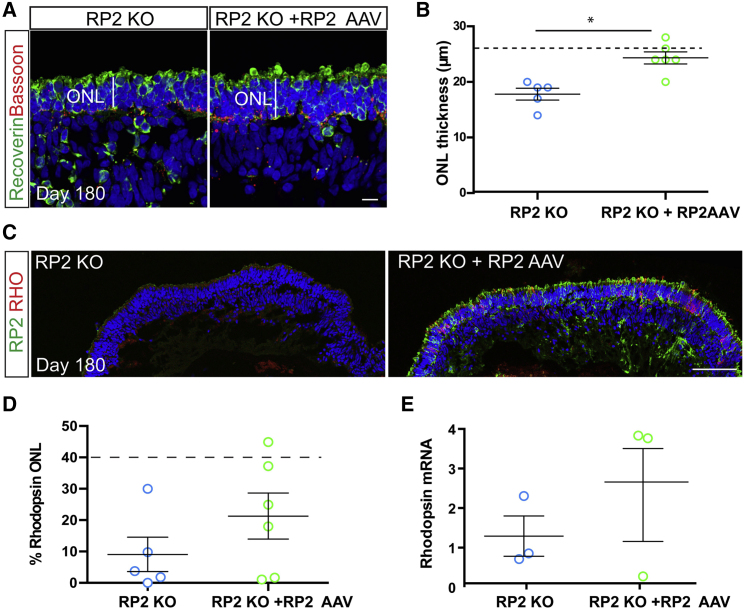
AAV2/5-Driven RP2 Overexpression Rescues Photoreceptor Survival (A) ICC of retinal organoid ONL with recoverin (green) and Bassoon (red) in RP2 KO control and AAV RP2-treated retinal organoids showing improved ONL thickness in the AAV-treated group. Scale bar, 10 μm. (B) Quantification of ONL thickness in control and AAV transduced retinal organoids (n = 5 RP2-KO, 6 RP2 KO + AAV independent organoids, ∗p = 0.016, mean ± SEM). Dotted line represents mean thickness in isogenic control parent cell line. (C) ICC showing RP2 (green) and rhodopsin (red) expression after transduction. Scale bar, 50 μm. (D) Quantification of ICC to assess rhodopsin-positive cells in control and RP2 AAV-transduced RP2 KO retinal organoids. Dotted line represents average control values (n = 5 RP2 KO, n = 6 RP2 KO + RP2 AAV independent organoids, p = 0.23; mean ± SEM). (E) qPCR for *RHO* mRNA levels in control and RP2 AAV-treated RP2 KO retinal organoids (n = 3 independent organoids). Mean ± SEM.

To investigate if the increase in RP2 levels was altering the degenerative phenotype of the ROs, ONL thickness and rhodopsin immunoreactivity were compared in RP2 AAV-transduced versus untransduced RP2 KO ROs at D180 (Figures 5A and 5C). The ONL was stained with recoverin and Bassoon (Figure 5A). Measurement of the ONL revealed that AAV-RP2-transduced RP2 KO organoids had a significantly thicker ONL than non-transduced controls at D180 (p ≤ 0.01, Figure 5B) with increased numbers of photoreceptors (Figure S5). The ONL thickness post-transduction was similar to that observed for the isogenic control cells at D180, suggesting near complete rescue. In most, but not all, transduced organoids the percentage of rhodopsin-positive cells was above the average for non-transduced RP2 KOs suggesting that AAV RP2 expression could restore rhodopsin immunoreactivity (Figure 5D). This was also observed at the mRNA level, with up to a 3-fold increase in *RHO* level in transduced ROs; however, one transduced RO showed no response at the level of rhodopsin expression (Figure 5E). The percentage of cone arrestin-positive cells was also reduced following AAV transduction, but *ARR3* mRNA was unchanged (Figure S5).

## Discussion

Here, we describe an *in vitro* model of RP2 XLRP and show that AAV gene augmentation of RP2 can successfully reverse measurable and clinically relevant disease phenotypes. The combination of iPSC reprogramming, CRISPR gene-editing technology, and AAV gene delivery has enabled a side by side comparison of RP2 KO, isogenic controls, and XLRP patient ROs, to obviate some of the inherent variation in iPSC-derived organoid models.

Differentiation of iPSCs presents an opportunity to probe genetic disease mechanisms in the cells and tissues that are affected by specific genetic changes. This is particularly useful in the case of ubiquitously expressed genes, such as *RP2*, that have disease pathology restricted to a specific tissue, such as the retina. Here, we used iPSC reprogramming technology in combination with CRISPR/Cas9 gene editing to probe disease mechanisms in *RP2* XLRP.

All of the RP2-deficient cell lines used in this study successfully developed 3D ROs with advanced morphological features, including lamination and formation of rod and cone photoreceptors. Strikingly, we observed a significant decline in photoreceptor cell numbers, specifically rhodopsin-positive rods, and significantly thinner ONL after 150 days of differentiation in all RP2-deficient lines. Widespread rhodopsin expression is one of the later events in RO development, in keeping with the developmental time line *in vivo*; however, the presence of other late-emerging cell markers in the RP2 KO and patient cell lines, such as cone arrestin-positive cone photoreceptors, PKCα-positive bipolar cells, and OS structures, together with the persistence of this rhodopsin-deficient phenotype up to 300 days of differentiation suggests that this is not merely a case of developmental delay.

This phenotype has not been reported in any existing animal models of RP2 XLRP (Li et al., 2013, Zhang et al., 2015), suggesting that this is either specific to human retina, or to the *in vitro* organoid system. Despite the relative stability of ROs in comparison with *ex vivo* retinal explant culture, which can be maintained for around 14 days (Johnson and Martin, 2008), it is likely that the conditions for retinal cell maintenance in culture are suboptimal. The absence of an opposing RPE monolayer, nutrient and oxygen deprivation through the lack of vascular blood supply, or the lack of connectivity for the inner retinal neurons, for example, may be sources of stress that accelerate the onset of disease phenotypes. This may go some way to explain the early “age” of detectable changes relative to mouse and human RP2 XLRP *in vivo*. In RP2 KO mice, ONL thinning is detectable only at 5 months of age in one model (Li et al., 2013), whereas almost no ONL thinning was observed at 12 months of age in another model (Zhang et al., 2015). Here, we detected measurable differences in photoreceptor survival at the onset of rod differentiation and rhodopsin expression. Cell death is an important, non-pathological process during retinal development (Vecino et al., 2004), but is increased during differentiation in the photoreceptor cell layer of RP2-deficient ROs, linking rod maturation temporally with rod cell death in this model. Interestingly, ROs from an individual with a mutation in *RPGR*, the other major form of XLRP, also showed signs of photoreceptor cell death at around 150 days of RO differentiation, which was rescued by CRISPR-mediated repair of the RPGR mutation in the iPSC (Deng et al., 2018). The induction of p53 pathways was also observed in the RPGR retinal organoid model (Deng et al., 2018), suggesting that this could be a common pathway in both forms of XLRP in human ROs. Therefore, photoreceptor cell death appears to be a feature in ROs for some forms of inherited retinal disease, but the precise mechanisms and whether there is an opportunity to inhibit the cell death process will require further investigation.

AAV is a highly efficient method of delivering therapeutic genes to photoreceptor cells and AAV2/5 has been shown to effectively transduce photoreceptors in a variety of species *inter alia* mice (Palfi et al., 2012), non-human primates (Boye et al., 2012), and human retinal explants (Wiley et al., 2018). The use of AAV in human ROs is currently limited, but studies have reported varying efficiency of transduction, which could be attributed to age of treatment, time of harvesting, vector tropisms, viral titer, the promotor used, and the sensitivity of detection of the transgene, e.g., antibody versus intrinsic fluorescence of a reporter (Garita-Hernandez et al., 2020, Gonzalez-Cordero et al., 2018, Quinn et al., 2019). Here, we demonstrate highly efficient transduction using an AAV2/5 vector with RP2 under the control of a CAG promoter. The AAV appears to deliver RP2 more or less exclusively to the photoreceptor cells despite the use of a ubiquitous promotor. This may be due to a combination of vector tropisms and the organization of the organoid, where the photoreceptor layer is outermost and, therefore, in contact the viral particles in solution. In addition, the OLM may prevent diffusion of AAV into the inner retinal layers. The interaction of AAV and photoreceptors in this system is analogous to the situation following subretinal delivery. Indeed, in a human treatment context, the optimal route of administration for this vector would be subretinal injection. Although intravitreal injection is less invasive, most AAV serotypes cannot transduce photoreceptors efficiently when administered intravitreally.

AAV RP2 was delivered at D140, before the onset of the increase in photoreceptor TUNEL reactivity and ONL thinning, but late enough to ensure efficient uptake, following reports of inefficient transduction at earlier time points (Gonzalez-Cordero et al., 2018). Not only was RP2 efficiently expressed at the mRNA and protein level, but it was also able to rescue the ONL-thinning phenotype in RP2 KO ROs implying a protective effect of RP2 overexpression in photoreceptor cells. Interestingly, although RP2 was expressed at over 55-fold the endogenous level, we did not observe any overt deleterious effects. In a previous study, some toxicity was observed with RP2 overexpression in mice 3 months after administration of the highest dose of AAV-RP2 vector (1 × 10^9^ viral genomes [vg]/eye), although the level of RP2 overexpression in transduced mouse eyes was not defined (Mookherjee et al., 2015). As RP2 is a GAP for ARL3, it is possible that increasing RP2 to a high level would inhibit the production of ARL3-GTP and mimic the effect of loss of the ARL3 GEF, ARL13b, which causes Joubert syndrome; however, this might not occur in the cellular context because of the spatial separation of the proteins, with RP2 predominantly at the base of the cilium and ARL13b in the axoneme, which leads to a gradient of ARL3-GTP in the cilium. Despite very high levels of RP2, once ARL3 is trafficked into the cilium it can be converted to ARL3-GTP by ARL13b; however, this would not exclude other activities of overexpressed RP2 from disrupting homeostasis. Therefore, additional studies evaluating the therapeutic index for AAV-RP2 therapies in ROs, and/or the primate eye, would be of value, before translation to the clinic.

Although RP2 is expressed in multiple retinal cell types, it remains to be established whether optimal rescue of the RP2 disease phenotype may require expression of a replacement *RP2* gene in other retinal cell types, such as the RPE. The use of a ubiquitous promoter in the current study should, in principle, enable expression of the therapeutic gene in multiple cell types. Additional studies will be required to fully elucidate the requirement, or otherwise, for RP2 in the RPE, to develop optimal RP2 gene therapies. In contrast to the current study, Mookherjee et al. (2015) used the photoreceptor-specific rhodopsin kinase promoter to drive expression of the RP2 gene and achieved a partial rescue of the cone phenotype in a null RP2 mouse model but had no beneficial effect on the rod phenotype.

This study provides insights into the use of ROs for retinal disease modeling, with a phenotype related to loss of RP2 that is unique to the human retina cell culture model. Importantly, the photoreceptor cell death in the RP2-deficient organoids correlates with the timing of rod cell maturation and rhodopsin expression. Furthermore, we highlight how the use of AAV for the restoration of RP2 expression can reverse this phenotype, and as such could be further investigated as a potential therapeutic avenue for the treatment of XLRP.

## Experimental Procedures

### Reprogramming and Gene Editing

Following informed consent, a skin biopsy was obtained from study participants to obtain dermal fibroblasts. The study followed the tenets of the Declaration of Helsinki and was approved by the Moorfields Eye Hospital and Royal Victoria Eye and Ear Hospital Dublin Research Ethics Committees. iPSCs were generated from two unrelated R120X individuals and control fibroblasts (BJ fibroblast ATCC CRL-2522) as described previously (Schwarz et al., 2015). RP2 KO iPSCs were produced by simultaneous reprogramming and gene editing using a method described previously (Howden et al., 2015). Guide RNAs were designed to target exon 2 of *RP2* (see Table S1 for sequences), and were cloned into the pSpCas9(BB)-2A-Puro (PX459) V2.0 plasmid (Addgene plasmid no. 62,988) according to a previously described protocol (Ran et al., 2013). iPSC clones were manually isolated, genomic DNA extracted (Promega), and PCR amplified with primers designed around the target site (Table S1). iPSC clones were analyzed by Sanger sequencing to confirm *RP2* gene disruption. Off-Spotter (https://cm.jefferson.edu/Off-Spotter/) was used to predict off-targets for the selected gRNA and the top 10 off-targets were assessed with Sanger sequencing, which detected no changes (Figure S1D; Table S1). In addition, all DE genes from the RNA-seq analysis were cross-referenced with off-target predictions and a further eight potential off-target sites with four or five mismatches were analyzed (Figure S1E; Table S1). These showed no sequence changes.

### Differentiation of iPSCs to ROs

RO differentiation was carried out as described previously (Nakano et al., 2012, Zhong et al., 2014). In brief, iPSCs were grown to near confluence in E8 medium before detaching colonies in gentle dissociation buffer (STEMCELL Technologies) to form embryoid bodies (EBs). EBs were transitioned to neural induction medium in the presence of blebbistatin, before plating down at a density of approximately 20 EBs per cm^2^. Emerging transparent pouches of neuroepithelium were isolated using a needle and cultured in suspension in retinal maturation media +0.5 μm retinoic acid up to D140, after which retinoic acid was removed. Alternatively EBs were generated by single-cell dissociation and forced aggregation in 96-well V-bottomed plates and cultured in suspension thereafter (Nakano et al., 2012). Days of culture are ±3days.

### AAV Production

The RP2 replacement vector comprising a CAG promoter, human RP2 CDS, RP2 3′ UTR, including an RP2 poly(A) sequence, and a minimal rabbit β-globin poly(A), was synthesized by GeneArt (Life Technologies) and cloned into pAAV-MCS using flanking NotI sites. More detail of sequences is in the Supplemental Information. Recombinant AAV2/5 viruses were generated by helper virus-free, triple transfection (Xiao et al., 1998). Human embryonic kidney cells (accession number CRL-1573; ATCC, USA) were transfected with pAAV-RP2, pRep/Cap5 (Hildinger et al., 2001) and pHelper (Agilent Technologies, USA) at a ratio of 1:1:2, as described previously (O'reilly et al., 2007). Seventy-two hours after transfection, AAV particles were purified from the clarified lysate by differential precipitation with polyethylene glycol followed by cesium gradient centrifugation (Ayuso et al., 2010). AAV-containing fractions were dialyzed against PBS supplemented with Pluronic F68 (0.001%; Bennicelli et al., 2008). Genomic titers (vg/mL) were determined by quantitative real-time PCR (qPCR; Rohr et al., 2002).

### AAV Treatment

RP2 AAV was prepared to a final titer 4.73 × 10^12^ vg/mL. At 140 days, organoids with brush borders visible by light microscopy, which show inner segment and OS development, were transferred to the well of 96-well plate and incubated with 1E11 viral genomes in 75 μL media for 8 h before topping the media up to 200 μL. Followed by 50/50 medium changes every 2 days until day 180.

### RNA Extraction and qPCR

ROs or half ROs were subjected to RNA extraction using an RNeasy MicroKit (QIAGEN) and cDNA synthesis was performed using a Tetro cDNA synthesis kit (Bioline). qPCR was carried out on an Applied Biosystems 7900HT Fast Real-Time PCR system using the SYBR Green method using 1 μL cDNA per triplicate. Data from the qPCR were normalized to the geometric mean of the expression of two internal reference genes in each sample. *POLR2A* and *MAN1B1* were chosen due to their consistency across the sample groups, which was determined using the GeNorm algorithm (Vandesompele et al., 2002). Primers were designed to cross exon boundaries (see Table S2).

### RNA-Seq

Three ROs from BJ controls and R120X cell lines and two ROs from the RP2 KO cell line were harvested at D150. The RNA was extracted with the RNeasy micro kit (QIAGEN) following the manufacturer’s instructions, followed by paired-end sequencing at 100 million read depth for each sample (Illumina, Otogenetics, Atlanta, GA, USA). Raw.fastq sequences were cleaned from any residual sequencing adapter using cutadapt with parameters -m 20 and -e 0.1. Fragments were then aligned to the human genome (build 38, Ensembl version 92) using STAR and genes counted using featurecounts.

Differential expression analysis was then performed using the DESeq2 pipeline. Initial inspection of the dataset showed biases, which were blind estimated and corrected using the sva package (DESeq2 manual). The differential expression analysis showed that 1,141 genes were differentially expressed between the R120X ROs and the control, whereas 979 genes were differentially expressed between the RP2 KO ROs and the isogenic control and 328 genes were common to the 2 RP2-depleted backgrounds. Of these, 232 genes were consistently upregulated and 56 were downregulated. The list of DE genes is shown in Table S3. Finally, we used the web platform Enricher to source any significant pathway (https://amp.pharm.mssm.edu/Enrichr/). According to the KEGG there was a significant upregulation of the “p53 signaling pathway” in the RP2 KO and RP2-R120X retina (Figure S3).

### Immunofluorescence

ROs were either fixed whole or bisected under a dissecting microscope using micro scissors (Fine Science Tools) with the other half processed for RNA extraction. ROs or half ROs were fixed in 4% paraformaldehyde at 4°C for 20–30 min before cryoprotection by immersion overnight in 30% sucrose/PBS. Following orientation under a dissecting microscope in OCT they were frozen and cryosectioned into 6-μm sections. For ICC, slides were blocked in 10% donkey serum, 0.1% Triton X for 1 h before incubation with primary antibodies for 2 h (see Table S4 for primary antibodies) and donkey anti-rabbit or mouse Alexa Flour 488 or 555 secondary antibodies for 1 h (Thermo Fisher). Nuclei were visualized using DAPI (2 μg/mL) staining for all images.

### Imaging

All images were obtained using a Carl Zeiss LSM700 or LSM 710 laser scanning confocal microscope. Images were exported from Zen imaging software and prepared using Adobe Photoshop and Illustrator CS4. All measurements were performed in Fiji (Schindelin et al., 2012). For quantification of TUNEL, rod and cone cell numbers, and ONL thickness measurements, the tilescan function was used to obtain images of whole organoid sections at 40× magnification (more detail in Supplemental Information). Individual rod-, cone-, and TUNEL-positive cell numbers in the ONL were manually counted across the whole section. ONL thickness was calculated by measuring the length and area of the whole recoverin-positive ONL in the DAPI channel using Fiji.

### Electron Microscopy

ROs were fixed overnight in 3% glutaraldehyde and 1% paraformaldehyde buffered to pH 7.4 with 0.08 M sodium cacodylate-HCl buffer. After rinsing in 0.1 M sodium cacodylate-HCl buffer (pH 7.4) twice for 5 min, the ROs were post-fixed in 1% aqueous osmium tetroxide for 2 h, dehydrated by passage through ascending alcohols (10-min steps, 1× 50%–90% and 3% × 100%) and two changes of propylene oxide, and infiltrated overnight with a 1:1 mixture of propylene oxide:araldite on a rotator. Finally, ROs were infiltrated with araldite resin over 4–6 h and embedded in fresh resin, which was then cured by overnight incubation at 60°C. Semithin sections (0.75 μm) were stained with a 1% mixture of toluidine blue-borax in 50% ethanol, and ultrathin sections were contrasted with Reynolds lead citrate for imaging in a JEOL 1010 TEM operating at 80 kV. Images were captured using a Gatan Orius CCD camera using Digital Micrograph software.

### Statistical Analysis

Statistical analysis was carried out on GraphPad Prism. Data were subjected to two-way ANOVA and/or by multiple t tests using the Holm-Sidak method, with α = 5.00%. Each time point was analyzed individually, without assuming a consistent standard deviation (SD). Significance was determined at a p value of < 0.05.

### Data and Code Availability

The RNA-seq data are available at GEO: GSE148300.

## Author Contributions

A.L., K.J., C.S., D.O., A.B.P., N.S., R.G., M.J.H., N.E.S., A.P., and N.C. performed the experiments and/or analyzed the data. A.L., K.J., C.S., D.O., N.E.S., G.J.F., A.J.H., and M.E.C. conceived the hypothesis and designed the experiments. A.L., K.J., C.S., G.J.F., A.J.H., and M.E.C. drafted the manuscript. All authors edited the draft manuscript.

## Supplemental Information

Document S1. Supplemental Experimental Procedures, Figures S1–S5, and Tables S1, S2, and S4

Table S3. List of DE Genes from D150 RNA-Seq Analyses, Related to Figure 2

Document S2. Article plus Supplemental Information

## Footnotes

Supplemental Information can be found online at https://doi.org/10.1016/j.stemcr.2020.05.007.
