# Supplementary material for: Modeling and Rescue of RP2 Retinitis Pigmentosa Using iPSC-Derived Retinal Organoids
Source: Stem Cell Reports. 2020 Jun 11;15(1):67–79. doi: 10.1016/j.stemcr.2020.05.007 (PMC7363745; doi:10.1016/j.stemcr.2020.05.007)
Supplement: Document S1. Supplemental Experimental Procedures, Figures S1–S5, and Tables S1, S2, and S4 [file mmc1.pdf]

Stem Cell Reports, Volume *15*

Supplemental Information

Modeling and Rescue of RP2 Retinitis Pigmentosa Using iPSC-Derived Retinal Organoids

Amelia Lane, Katarina Jovanovic, Ciara Shortall, Daniele Ottaviani, Anna Brugulat Panes, Nele Schwarz, Rosellina Guarascio, Matthew J. Hayes, Arpad Palfi, Naomi Chadderton, G. Jane Farrar, Alison J. Hardcastle, and Michael E. Cheetham

Supplemental Information

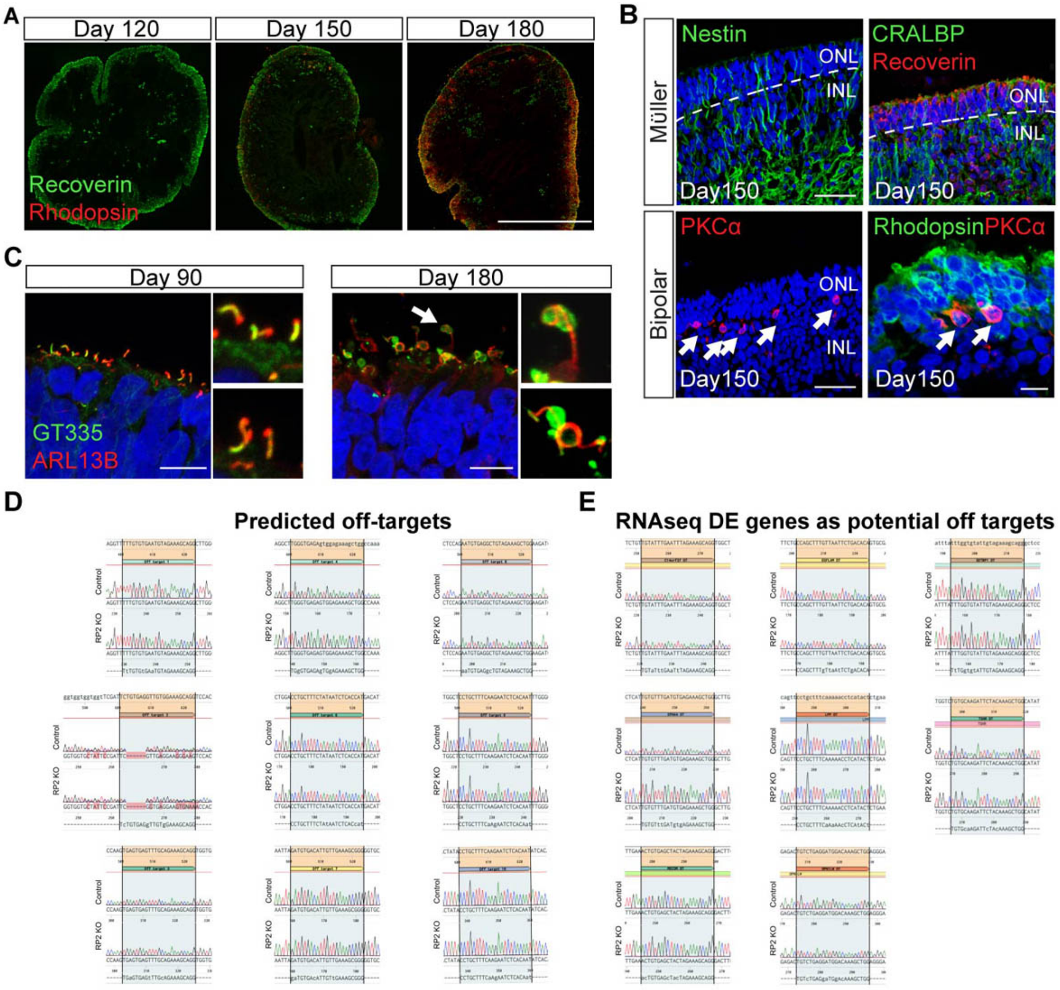

Figure S1. ***Control retinal organoid differentiation and lack of off-target editing in the RP2 KO.*** Related to Figure 1 and experimental methods.

A. ROs show increased rhodopsin expression between D150 and D180 Scale bar =500 µm.

B. ROs contain Muller glia, stained with nestin (green) and CRALBP (green) and bipolar cells, stained with PKCα Scale bars left = 50 µm; lower right = 10 µm**.**

C. Apical cilia in ROs stained with ARL13b (red) and GT335 (green) Scale bars = 10 µm.

D. Sanger sequencing results of Top 10 predicted off-targets for RP2 gRNA2. No mutations were observed in any of the predicted off-target sites. Off target 2 happens to occur in a region where the control line has a naturally occurring variation. Lower case letters in the off-target sequences below the traces indicate the sites at which the off-targets vary from RP2 gRNA2.

E. Sequencing results of RNAseq DE genes with possible similarity to the gRNA sequence. Sanger sequencing revealed that none of these sites showed any editing by off-target Cas9 activity.

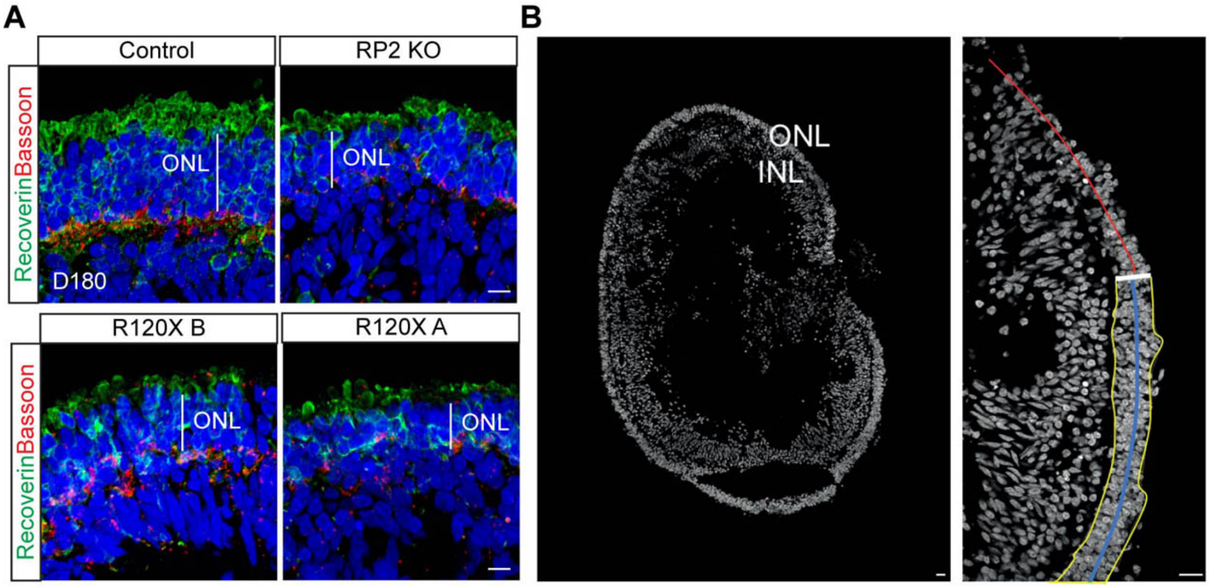

Figure S2. ***Thinning of the ONL in RP2 null cells.*** Related to Figure 2.

A. Representative images showing reduced ONL thickness in RP2 KO, R120X A and R120X B ROs. The Control and RP2 KO images are also shown in Figure 2. Scale bar = 10 µm.

B. Tilescans of retinal organoid to illustrate the method of ONL measurement. Whole organoid (left panel) close up of ONL (right panel). The area of the ONL is enclosed within the yellow marked area and the length of ONL indicated by the blue line. The red line indicates an area of non-photoreceptors that were not included. Scale = 20µm

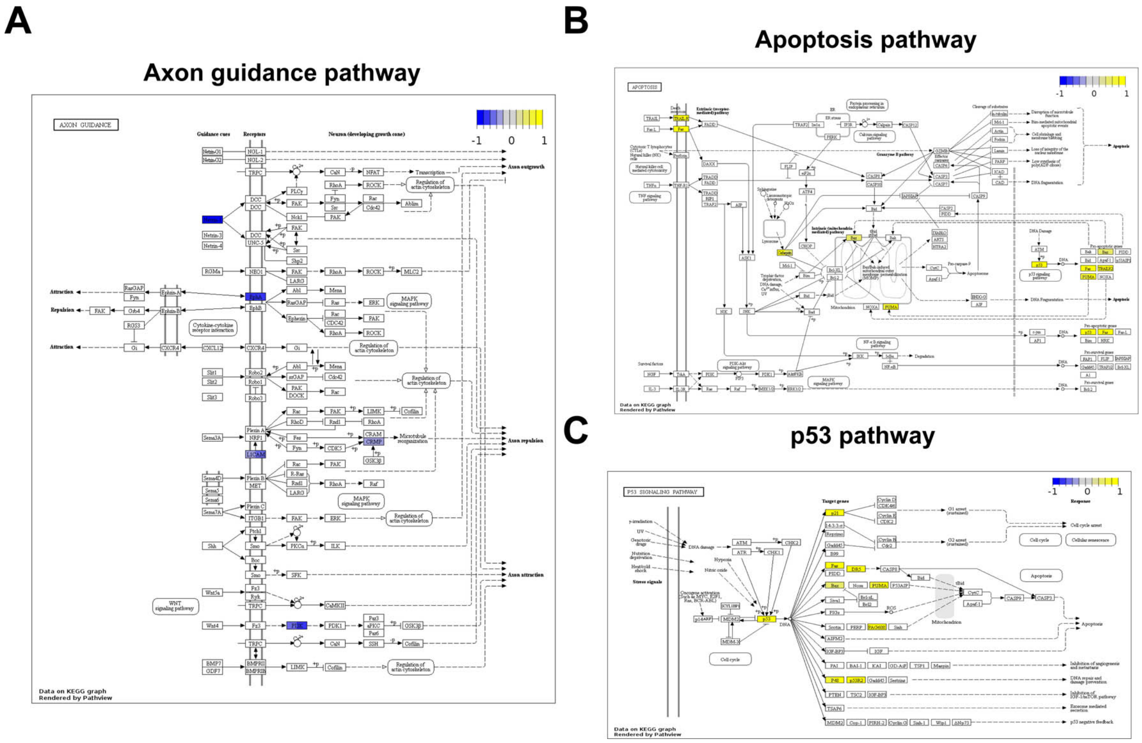

Figure S3. ***KEGG analyses of shared DE genes between RP2 nulls and control.*** Related to Figure 2.

A. KEGG analyses of RP2 KO and RP2 R120X common downregulated DE genes compared to control ROs at D150 in 'axon guidance' pathways.

B. KEGG analyses of RP2 KO and RP2 R120X common DE genes that are upregulated compared to control ROs at D150 in the 'apoptosis' pathway.

C. KEGG analyses of RP2 KO and RP2 R120X common DE genes that are upregulated compared to control ROs at D150 in the 'p53' pathway

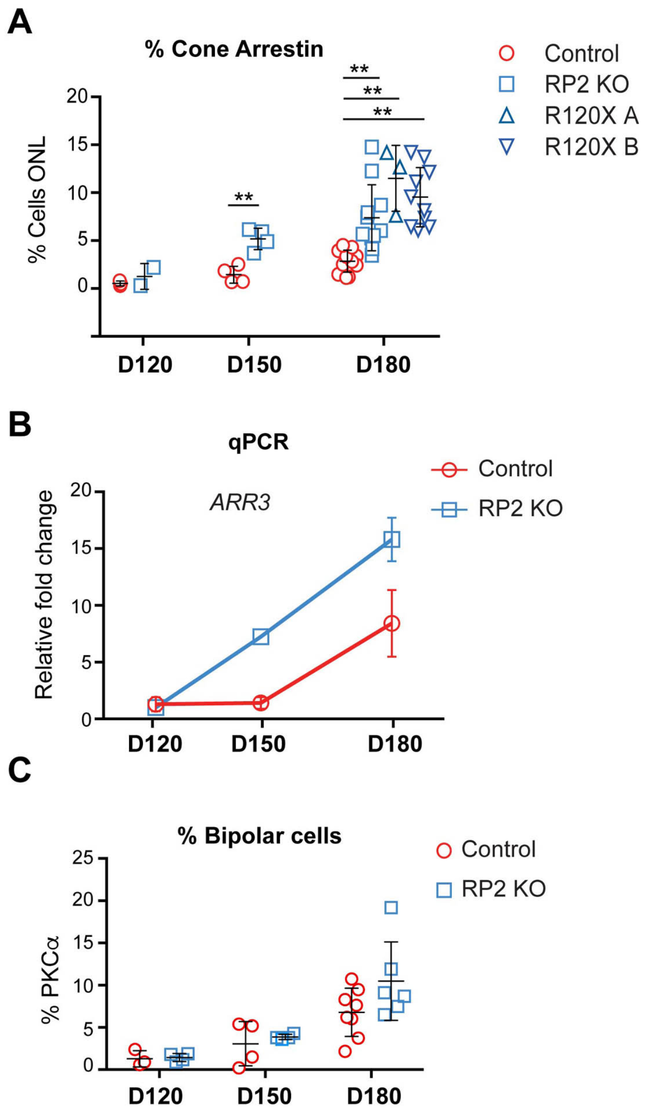

Figure S4. ***Cone photoreceptor and bipolar cells in RP2 null organoids.*** Related to Figure 3.

A. Percent of cells in ONL positive for cone arrestin immunostaining, Control (red circle; n=3, D120; n=4 D150 n=10 D180); RP2 KO (blue square; n=2, D120; n=4 D150 n=10 D180) R120X A (blue triangle; n=3 D180), R120X B (blue inverted triangle; n=10 D180); ** p<0.01 each n represents the mean from a full tilescan of an independent organoid. Mean ±SD

B. *ARR3* levels in retinal organoids. qPCR showing relative fold change in mRNA in control and RP2 KO retinal organoids at D120, D150 and D180 (n=3,3,4 independent organoids respectively; mean ±SEM).

C. Cell counts of bipolar cells (positive for PKCα) in control and RP2 KO ROs showed no significant differences in bipolar cell numbers at D120, D150 or D180 (controls n=3, 4 and 8 RP2 KO n=4, 4, 6, mean of tilescan of full independent organoids; mean ±SEM).

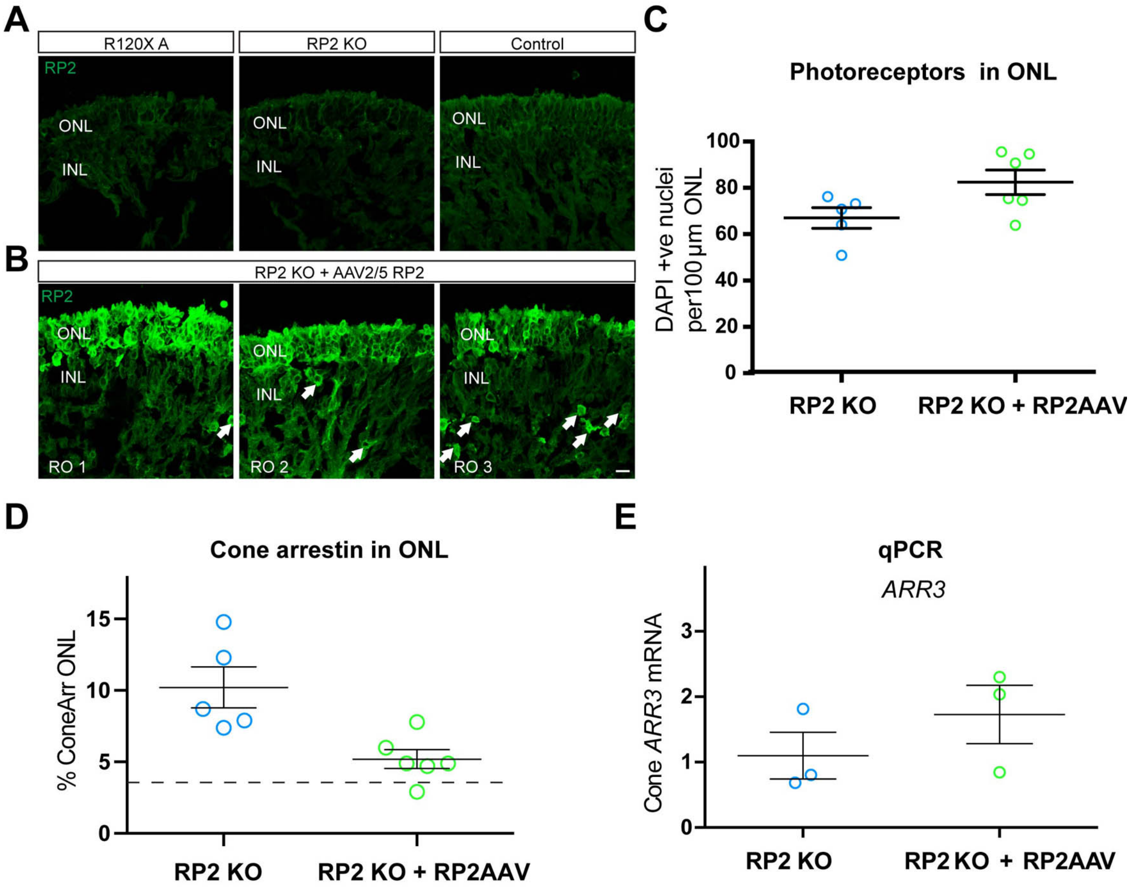

Figure S5. ***Transduction of RP2 KO organoids with RP2-AAV.*** Related to Figure 5.

A. ICC for RP2 in untreated D180 RP2 R120X A and RP2 KO and control ROs.

B. RP2 immunostaining after RP2-AAV transduction in 3 different organoids (RO1, RO2 and RO3). The few strongly positive cells for RP2 outside of the ONL are indicated with arrows. Scale bar = 10 µm.

C. The number of photoreceptors in the ONL. Mean of nuclei in the ONL per 100 µm (n=5 RP2 KO and n=6 RP2 KO + RP2AAV independent organoids for each condition; p=0.058; mean ±SEM).

D. Percentage of cells in the ONL that were positive for cone arrestin staining in untreated and after RP2-AAV transduction at D180, the dotted line indicates the mean percent in controls (RP2 KO n=5, RP2+RP2AAV n=6 independent organoids; p=0.055; mean ±SD).

E. *ARR3* levels in retinal organoids following RP2-AAV transduction. qPCR showing relative fold change in mRNA at D180 (n=3 independent organoids for both conditions; mean ±SEM).

Table S1. gRNA, Primer and Off-target sequences. Related to Figures 1, 4, and S1

Target	Forward	Reverse
**gRNA sequence for RP2 exon 2 CRISPR**		
RP2 gRNA2	TGTGTGAGATTGTAGAAAGC**TGG**	
**PCR primer sequences**		
RP2 exon2	CCAAAAGACTACATGTTCAGTGGA	GCATGATTCATCGCTGCTCT
**Off-target PCR Primer sequences**		
OT1 – Chr13 + 99786425 - 99786447	GGTCAGGGCCAGCACATAAT	TAGCTAATGTGCCCTCAGCG
OT2 - Chr 6 + 38143360 – 38143382 (BTBD9 pre-mRNA)	ACCACGAGGGAGTTAGAGGA	AGCCACATGAGGTTGAAGTCC
OT3 - Chr 9 + 91974085 – 91974107 (SECISBP2 pre- mRNA)	AGTTCTTGCCTGTGTGTCTGT	TAGCAGAACCCCCACTACGC
OT4 - Chr 7 + 157998199 – 157998221 (PTPRN2 pre-mRNA)	TAGCCAAAGCTCTTGCTTCAGT	GTGCCCTATCCTACTTGGCTG
OT5 - Chr 3 – 132906887 -132906909 (TMEM108 pre-mRNA)	CTGTCAAGGAAGGAAGGGAGG	GGAGAAAGGGCCCCCTATAA
OT6 - Chr 5 – 116400519 - 116400541	AACTGTGTCCCACAATAAGGGG	TGCTGGGACCTCCTCAAATTC
OT7 - Chr X + 119452379 - 119452401	AAGCCCACAGGAACTGGGTT	AAACCAAAGTAGGTGGGTCCT
OT8 - Chr 10 + 22037281 - 22037303	ATTCTGCCGGGGTTTAGAGC	AGCAAGGTGAACTGACACTGG
OT9 - Chr 1 – 23887266 - 23887288	AGTAGCTCCAGCTCCATCTGA	ATCTTTCTTGGGCCACAGAGC
OT10 - Chr 11 – 109695393 - 109695415	AAAATGGCTATGCAGCACAAGTT	TTAGCTTCTCCCTTACACCTGAAC
**RNAseq DE gene potential off-target PCR Primer sequences**		
C14orf37	ACTGGGCTTTGAAAGATGGAGA	AGGCACTCAAATGGTGGAGC
EGFLAM	TAGGGCTTTGCACATCGCA	CGGGTTTAGTGGGAAGGCAG
EPHA4	CCTTGTCGATCTTCCACTCCC	ATGACGTGTGGAGCACTGAC
LPP	TCCGACTTTGCCTTCCTTCT	CTCTGTGGCACCTATCACAACT
MECOM	AGCTGCCATTGCTTTTTCTCA	TGCTTAGGAAACACACGCCT
OPN1LW	TGGGTAGTGGTCTTCCCCTC	AGCCCATTTGTGAAGACGGA
SETBP1	TTTCATGCCAGTGACCTTCG	TTGCTCATTGACTTTTGACATGGG
TSHR	AGCCACAACCACTCTTGACT	TGACTGGTTTTCAGGTGCTAGAT

Table S2. Primer sequences for qPCR. Related to Figures 3, 4, 5 and S4, and S5

qPCR primer sequences		
**Target**	**Forward**	**Reverse**
RHO	GGTGGTGTGTAAGCCCATGA	CCTCGGGGATGTACCTGGAC
GNAT1	CTGCTCACTCTGTCCCTTCG	TTGACGATGGTGCTCTTCCC
RP2 EX1-2	GACCATGGGCTGCTTCTTCT	ACTGTTGTCCTGCTACCGTC
ARR3	CATGTGCCTTTCGCTATGGC	TCAATCCCACAGGGCTTTCC
MAN1B1	ACCGTGGAGAGCCTGTTCTA	GTTTGGGTCATCGGAGAAGA
GAPDH	CCCCACCACACTGAATCTCC	GGTACTTTATTGATGGTAC
POLR2A	GCGGGGTGAAGTGATGAAC	ATCATCGGGATGGGTGCTGT

Table S3. List of DE genes from D150 RNAseq analyses. Related to Figure 2.
See excel file Supplementary Table S3.xls.

Table S4. List of antibodies used. Related to all figures.

Marker	Species	Dilution	Supplier	Catalog number
Oct4	Rabbit	1:1000	Abcam	ab19857
Nanog	Mouse	1:1000	Thermo Invitrogen	MA-1-017
RP2 (western blot)	Sheep	1:2500	Sheep serum	References 1, 2
RP2 (ICC)	Rabbit	1:200	ProteinTech	14151-1-AP
Recoverin	Rabbit	1:500	Millipore	AB5585
Cone Arrestin (7G6)	Mouse	1:100	Gift from Wolfgang Baehr	
Rhodopsin (4D2)	Mouse	1:500	Millipore	MABN15
F-actin	AlexaFluor 488 Phalloidin		Thermo Invitrogen	A12379
TOM20	Mouse	1:150	Santa Cruz	SC17764
GT335	Mouse	1:800	Adipogen Life Sciences	AG-20B-0020
Arl13B	Mouse	1:1000	Abcam	ab136648
Bassoon	Mouse	1:500	Enzo	SAP7F407
Nestin	Mouse	1:200	Abcam	ab22035
CRALBP	Mouse	1:300	Thermo Invitrogen	MA1-813
PKCα	Rabbit	1:200	Abcam	ab32376

Supplemental Experimental Procedures

Gene editing off targeting assessment

Possible off-targets for the RP2 gRNA2 were predicted using the Off-Spotter online tool (https://cm.jefferson.edu/Off-Spotter/). Sequences from the RP2 KO line and the isogenic control line were aligned using Benchling (www.benchling.com). No mutations were observed in any of the predicted off-target sites. Off target 2 (Figure S1) happens to occur in a region where the control line has a naturally occurring variation. Primers for Off target 5 failed to amplify the DNA due to low complexity and high GC content in the target region. In addition, the list of DE genes generated by RNAseq analysis of RP2 KO vs control ROs was cross referenced with the list of Off-targets for RP2 gRNA2 (Off-spotter online tool; 431 off-targets predicted). Sanger sequencing revealed that none of these sites showed any editing by off-target Cas9 activity.

AAV production

The following sequences were used in the construct: CMV IE enhancer (661-1024 bp from GenBank: X03922), Ch-B-Actin proximal promoter/intron 1 (251-1542 bp of GenBank: X00182; 1538-39 bp AG was changed to CA); RP2 CDS (190-1270 bp of RefSeq: pNM_006915.2), RP2 3'UTR (1271-2440 bp of RefSeq: NM_006915.2), RP2 poly(A) (50341-50580 bp of RefSeq: NG_009107.1) and minimal rabbit B-globin poly(A) (Levitt et al., 1989)[3].

ONL measurement methods

Mean ONL measurements for control, RP2 KO and R120X patient derived ROs were assessed at three time points (D120, D150 and D180). Each data point represents the mean ONL measurement for an individual unique organoid. Mean ONL measurements were performed on confocal images taken with a 40x objective on a Zeiss LSM710. Organoids varied in size but approximately 12-20 images were acquired with the 40x objective so that the entire organoid section was imaged in a series of tiles. Each tile consisted of a maximum intensity projected Z stack. These tiles were then merged to produce a composite of the entire ONL in each organoid and processed in image J (FIJI). In mature ROs the ONL layer is easily determined by the organisation of nuclei in the DAPI channel, but this was also verified by examining the termination of photoreceptor marker staining (e.g. recoverin, rhodopsin, cone arrestin). Furthermore, between the densely packed nuclei of the ONL there is a physical gap to the INL (Fig. S2), this synaptic outer plexiform layer is labelled with bassoon in Fig 2A and S2. In imageJ (FIJI) the polygon selection tool was used to measure the area around the entire organised ONL in the DAPI channel (yellow line in Fig. S2B). In organoids where the full section was not organised into ONL/INL (e.g if an uncharacterised non-retinal area was present) the junction between retina and non-retina where the ONL tapers away was excluded in all cases (red line in Fig. S2B). In sections where 2 or more retinal 'lobes' were separated by non-retinal tissue, the lobes were measured separately and averaged into one number. The segmented line tool was used to draw a line through the centre of the selection area and the length (in pixels) measured (blue line Fig. S2B). The mean ONL thickness was then determined by division of the total area by the length. In this way deviations in ONL thickness throughout the organoid were accounted for. The final value (in pixels) was then converted to micrometers using for the scaling information from the acquisition format and 40x objective used.

TUNEL assessment method

The percentage of TUNEL positive nuclei in the outer nuclear layer for control, RP2KO and R120X patient derived organoids was derived over three time points, D120, D150 and D180 (Fig. 2D). Each data point represents TUNEL data from an individual unique organoid. TUNEL measurements were carried out on confocal images taken with a 40x objective on a Zeiss LSM710. Organoids varied in size but approximately 12-20 tiles at the 40x objective were acquired so that the entire organoid section was imaged. Each tile consisted of a maximum intensity projected Z stack. These tiles were then merged and opened in image J (FIJI). The area comprising ONL was determined as described above. DAPI nuclei numbers were counted manually in image J. Nuclei were counted in a representative area of ONL for each organoid and the number extrapolated based on total ONL area for each tile scan. TUNEL reactive nuclei within the predetermined ONL area were counted manually in the appropriate channel. The % TUNEL positive nuclei per organoid was then calculated. Across all data points the mean number of nuclei included per organoid was 1522 and the number of TUNEL positive nuclei counted was between 1 and 43.

Supplemental References

1. Chapple JP, Hardcastle AJ, Grayson C, Spackman LA, Willison KR, Cheetham ME (2000) Mutations in the N-terminus of the X-linked retinitis pigmentosa protein RP2 interfere with the normal targeting of the protein to the plasma membrane. Hum Mol Genet 9: 1919-26

2. Grayson C, Bartolini F, Chapple JP, Willison KR, Bhamidipati A, Lewis SA, Luthert PJ, Hardcastle AJ, Cowan NJ, Cheetham ME (2002) Localization in the human retina of the X-linked retinitis pigmentosa protein RP2, its homologue cofactor C and the RP2 interacting protein Arl3. Hum Mol Genet 11: 3065-74
3. Levitt, N., Briggs, D., Gil, A. & Proudfoot, N. J. (1989). Definition of an efficient synthetic poly(A) site. *Genes Dev,* 3**,** 1019-25.
