# Supplementary material for: Modeling and Rescue of RP2 Retinitis Pigmentosa Using iPSC-Derived Retinal Organoids
Source: Stem Cell Reports. 2020 Jun 11;15(1):67–79. doi: 10.1016/j.stemcr.2020.05.007 (PMC7363745; doi:10.1016/j.stemcr.2020.05.007)
Supplement: Document S2. Article plus Supplemental Information [file mmc3.pdf]

Stem Cell Reports
Article
OPEN ACCESS

Modeling and Rescue of RP2 Retinitis Pigmentosa Using iPSC-Derived Retinal Organoids

Amelia Lane,[1,3] Katarina Jovanovic,[1,3] Ciara Shortall,[2] Daniele Ottaviani,[1] Anna Brugulat Panes,[1] Nele Schwarz,[1] Rosellina Guarascio,[1] Matthew J. Hayes,[1] Arpad Palfi,[2] Naomi Chadderton,[2] G. Jane Farrar,[2,*] Alison J. Hardcastle,[1,*] and Michael E. Cheetham[1,*]

[1]UCL Institute of Ophthalmology, London, UK
[2]Smurfit Institute of Genetics, Trinity College Dublin, Dublin 2, Ireland
[3]Co-first author
*Correspondence: jane.farrar@tcd.ie (G.J.F.), a.hardcastle@ucl.ac.uk (A.J.H.), michael.cheetham@ucl.ac.uk (M.E.C.)
https://doi.org/10.1016/j.stemcr.2020.05.007

SUMMARY

RP2 mutations cause a severe form of X-linked retinitis pigmentosa (XLRP). The mechanism of *RP2*-associated retinal degeneration in humans is unclear, and animal models of *RP2* XLRP do not recapitulate this severe phenotype. Here, we developed gene-edited isogenic *RP2* knockout (RP2 KO) induced pluripotent stem cells (iPSCs) and *RP2* patient-derived iPSC to produce 3D retinal organoids as a human retinal disease model. Strikingly, the RP2 KO and *RP2* patient-derived organoids showed a peak in rod photoreceptor cell death at day 150 (D150) with subsequent thinning of the organoid outer nuclear layer (ONL) by D180 of culture. Adeno-associated virus-mediated gene augmentation with human RP2 rescued the degeneration phenotype of the RP2 KO organoids, to prevent ONL thinning and restore rhodopsin expression. Notably, these data show that 3D retinal organoids can be used to model photoreceptor degeneration and test potential therapies to prevent photoreceptor cell death.

INTRODUCTION

The reprogramming of patient-derived cells into induced pluripotent stem cells (iPSCs) has enabled the derivation and differentiation of a range of somatic cell types and has revolutionized our ability to study inherited disease (Takahashi et al., 2007). The differentiation of iPSCs toward retinal lineages has seen huge advances in recent years with the refinement of protocols for the generation of 3D retinal organoids (ROs) (Gagliardi et al., 2019; Nakano et al., 2012). Unlike previous models in 2D, these 3D structures contain photoreceptors with morphologically identifiable features; including, inner segments rich in mitochondria, rudimentary outer segments with connecting cilia, and synaptic pedicles, in addition to bipolar, Müller glia, ganglion and amacrine cells, synaptic layers, and an outer limiting membrane (OLM), arranged in retinal layers (reviewed in Capowski et al., 2019). These advanced models have proven to have many translational research applications, including transplantation studies (Gonzalez-Cordero et al., 2017; Shirai et al., 2016), retinal disease modeling, and testing the efficacy of potential therapies in human photoreceptor cells (Deng et al., 2018; Parfitt et al., 2016; Schwarz et al., 2017; Sharma et al., 2017). To date, however, they have not been used to model and rescue photoreceptor cell death.

Mutations in *RP2* account for approximately 15% of all cases of X-linked retinitis pigmentosa (XLRP) (Breuer et al., 2002; Hardcastle et al., 1999). RP2 is a GTPase-activating protein (GAP) for the small GTPase ARL3 (Veltel et al., 2008), which is also regulated by its guanine nucleotide exchange factor (GEF) ARL13B (Gotthardt et al., 2015). ARL13B is localized to the ciliary axoneme, whereas a pool of RP2 and ARL3 localize at the basal body and associated centriole at the base of photoreceptors (Evans et al., 2010; Grayson et al., 2002). ARL3, with its effectors (UNC119 and PDEdelta [PDED]) and GAP RP2, are thought to be important in the retina to traffic lipidated proteins, such as transducin, GRK1, and PDE6, to the photoreceptor outer segment (Ismail et al., 2011; Schwarz et al., 2012; Wright et al., 2011; Zhang et al., 2011; Zhang et al., 2015). *Rp2* knockout mice have a relatively mild phenotype compared with human disease. In one model mis-localization/absence of GRK1 and cone PDE6a was evident at 14 months (Zhang et al., 2015), whereas another model was reported to have rhodopsin and M opsin mis-localization at 2 months and outer nuclear layer (ONL) thinning at 5 months (Li et al., 2013). In contrast, the human phenotype is relatively severe with some patients experiencing macular atrophy in childhood (Jayasundera et al., 2010), highlighting the necessity for human retinal models of disease.

Currently there are no treatments for this condition, so there is a need to develop potential therapies. Characterization of iPSC-derived RPE and early-stage ROs from an individual carrying a nonsense mutation in *RP2* (c.358C > T, p.R120X) showed changes in Golgi cohesion, Gbeta trafficking in RPE, and ciliary trafficking of KIF7 in ROs (Schwarz et al., 2015, 2017). Furthermore, treatment with the readthrough drugs, G418 and/or Ataluren (PTC124),

This is an open access article under the CC BY license (http://creativecommons.org/licenses/by/4.0/).

could restore detectable full-length RP2 protein and rescue the Golgi cohesion and Gbeta mis-localization in iPSC-RPE and kinesin traffic in ROs. Gene therapy for other inherited retinal diseases using adeno-associated viruses (AAVs) has been shown to efficiently transduce photoreceptors and RPE following subretinal injection (Sarra et al., 2002) in animal models. There is a Food and Drug Administration- and European Medicines Agency-approved AAV-mediated ocular gene therapy (Russell et al., 2017), and a number of AAV-mediated ocular gene therapies are currently in phase I/II and III clinical trials (clinicaltrials.gov). AAV delivery of human RP2 to a mouse knockout model of RP2-XLRP preserved cone function, but had no effect on rod cell function and toxicity was observed at a higher viral dose (Mookherjee et al., 2015).

Here, we describe the temporal maturation of CRISPR gene-edited *RP2* knockout ROs relative to their isogenic control, in addition to ROs derived from two unrelated individuals with the same R120X nonsense mutation. These studies reveal that the loss of RP2 leads to rod photoreceptor degeneration that can be rescued by AAV delivery of RP2.

RESULTS

RP2 Knockout and *RP2* Patient iPSCs Develop Mature ROs

Fibroblasts from two unrelated individuals (R120X-A and R120X-B) carrying the nonsense mutation c.358C > T; p.R120X were reprogrammed into iPSCs by nucleofection (Okita et al., 2011; Schwarz et al., 2015). CRISPR/Cas9 with guides designed to target exon 2 of *RP2* were used to generate RP2 knockout iPSCs using a simultaneous reprogramming and gene-editing protocol (Howden et al., 2015). The location of the gRNA on exon 2 was selected due to its proximity to the c.358C > T p.R120X site, thus any knockout lines generated would closely mimic the consequences of this nonsense mutation. RP2 KO iPSC clones were identified through non-homologous end-joining-mediated creation of indels; one clone with an 8-bp deletion in exon 2 of *RP2* (RP2 c.371_378delAAGCTGGA; p.Lys124SerfsTer11) was selected for further study as it had the shortest frameshift extension before a premature stop codon. Western blotting of iPSCs showed efficient knockout, as no RP2 protein was detectable (Figures 1A and 1B).

The RP2 KO line, the non-edited isogenic control, and two R120X patient iPSC lines were differentiated into ROs over a period of 4–10 months using methods described previously (Nakano et al., 2012; Zhong et al., 2014) with slight modifications. Both methods produced biologically similar organoids that were developmentally and structurally comparable at the time points tested. By day 180 (D180) a transparent ONL with a brush-like border was visible by light microscopy (Figure 1C). Scattered rhodopsin-positive cells were first detectable in control organoids in the recoverin-positive ONL from D150 then increased in number over time as the ROs matured up to D180 (Figure S1A). By D180 all cell lines were able to generate ROs consisting of a laminated structure with a compacted ONL containing recoverin and cone arrestin-positive photoreceptors (Figure 1D), above an inner nuclear layer (INL) containing protein kinase C alpha (PKCα)-positive bipolar cells and CRALBP/Nestin-positive Müller glia (Figure S1B). In all ROs, the ONL terminated at the apical edge, with an OLM that was strongly immunoreactive for F actin (Figure 1D). Above the OLM, mitochondria (immunoreactive for TOM20) were enriched in globular inner segments. Immunostaining for the ciliary marker ARL13B and polyglutamylated tubulin (GT335) revealed the bulging shape at the tip of the photoreceptor connecting cilia, as they matured to form outer segment (OS) like structures from D150 onward (Figure S1C). RP2 could be detected in control ROs at the plasma membrane in all cells in the INL and ONL. In RP2 KO and R120X cell lines, RP2 immunoreactivity was absent by immunocytochemistry (ICC), although some background fluorescence was observed at the edges of the presumptive OS. qPCR was used to measure relative *RP2* mRNA expression in ROs at D180. The RP2 KO clone had only 20% *RP2* mRNA relative to its isogenic control, similar to the R120X patient ROs (n = 3) (Figure 1E), suggesting that the mutant allele transcript is subject to nonsense-mediated decay in all these ROs. Electron microscopy confirmed the presence of membranous-rich structures at the apical ciliary tip, reminiscent of early OS formation, in both control and RP2 KO ROs (Figure 1F). These rudimentary OS were often found detached from the body of the RO indicating the flexibility, or fragility, of these structures in the absence of RPE. Collectively, these results show that RP2 ablation does not prevent the differentiation of iPSC into photoreceptors bearing OS-like structures in 3D RO culture.

Loss of RP2 Leads to Photoreceptor Cell Death and ONL Thinning

Similar to the neural retina *in vivo*, the outermost cell layer of the ROs consists of a uniform compacted ONL, which terminates with photoreceptor synaptic pedicles that are separated from the inner retinal cells by a layer immunoreactive for synaptic structural protein Bassoon in the outer plexiform layer (Figure 2A). A reduction in the number of photoreceptor nuclei in the ONL, and thereby thickness, *in vivo* is a marker of photoreceptor cell degeneration. Therefore, ONL thickness was measured in isogenic controls and RP2 KO at D120, D150, and D180 (Figure 2B).

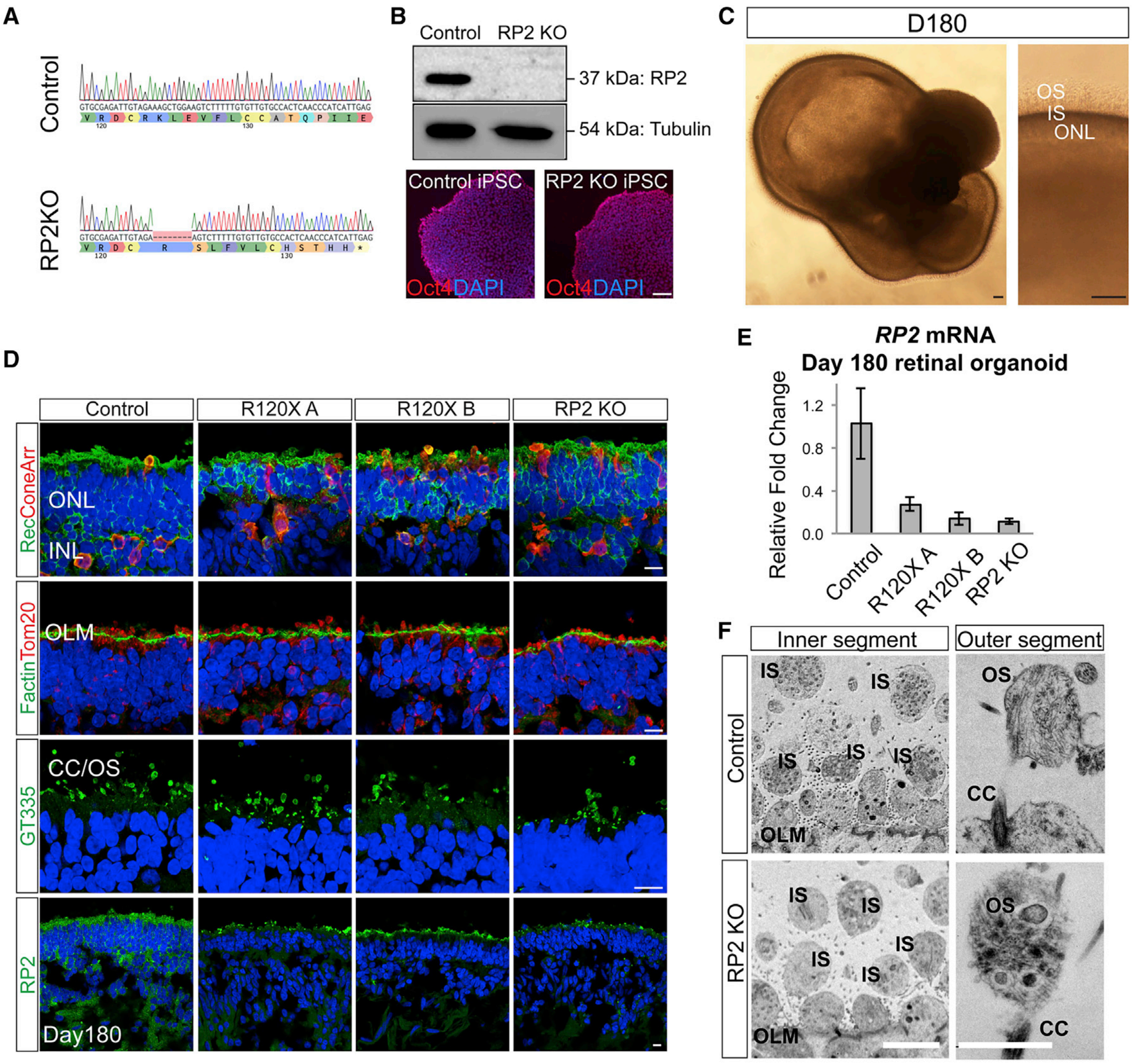

Figure 1. Retinal Organoids from R120X, RP2 KO, and Isogenic Control iPSCs

(A) Sanger sequence trace of edited RP2 KO iPSCs. CRISPR/Cas9 gene editing was used to create an 8-bp deletion in exon 2 of *RP2* by NHEJ.
(B) Western blot and immunocytochemistry (ICC) of control and RP2 KO iPSC. Scale bar, 50 μm.
(C) Retinal organoid morphology at D180, a layer of IS/OS is visible by differential interference contrast above the transparent ONL. Scale bar, 50 μm.
(D) ICC of retinal organoids at D180. Photoreceptors (recoverin and cone arrestin [coneArr]), outer limiting membrane (OLM, F actin) mitochondria (Tom20), and connecting cilia/OS (GT335) expression in the ONL of control and RP2 null iPSC retinal organoids. RP2 is expressed at the plasma membrane of cells in the ONL and inner nuclear layer (INL) in control organoids. Scale bar, 10 μm.
(E) qPCR of *RP2* mRNA in whole retinal organoids at D180 (n = 3 independent organoids). Mean ± standard error of the mean (SEM).
(F) Electron micrographs of control and RP2 KO retinal organoids at D180. Scale bars, 5 μm (left), 1 μm (right).

In control ROs, the average ONL thickness increased between D120 and D150 from 20 to 25 μm, then did not change significantly between D150 and D180. In contrast, in RP2 KO ROs the average ONL thickness decreased significantly between D150 and D180 ($p = 0.02$). Similarly, R120X ROs from both patients had significantly thinner ONLs at D180 compared with the control cell line ($p \leq 0.01$; Figures 2 and S2). This suggested that photoreceptor cell death might be occurring between D150 and D180 in the RP2 null cell lines.

To test this hypothesis further, we measured TUNEL reactivity across the ONL in RP2 KO and control ROs at D120, D150, and D180 (Figures 2C and 2D). A small percentage of TUNEL-positive nuclei were detectable in the photoreceptor ONL at all time points. In control ROs, TUNEL reactivity was not significantly different at D120, D150, or

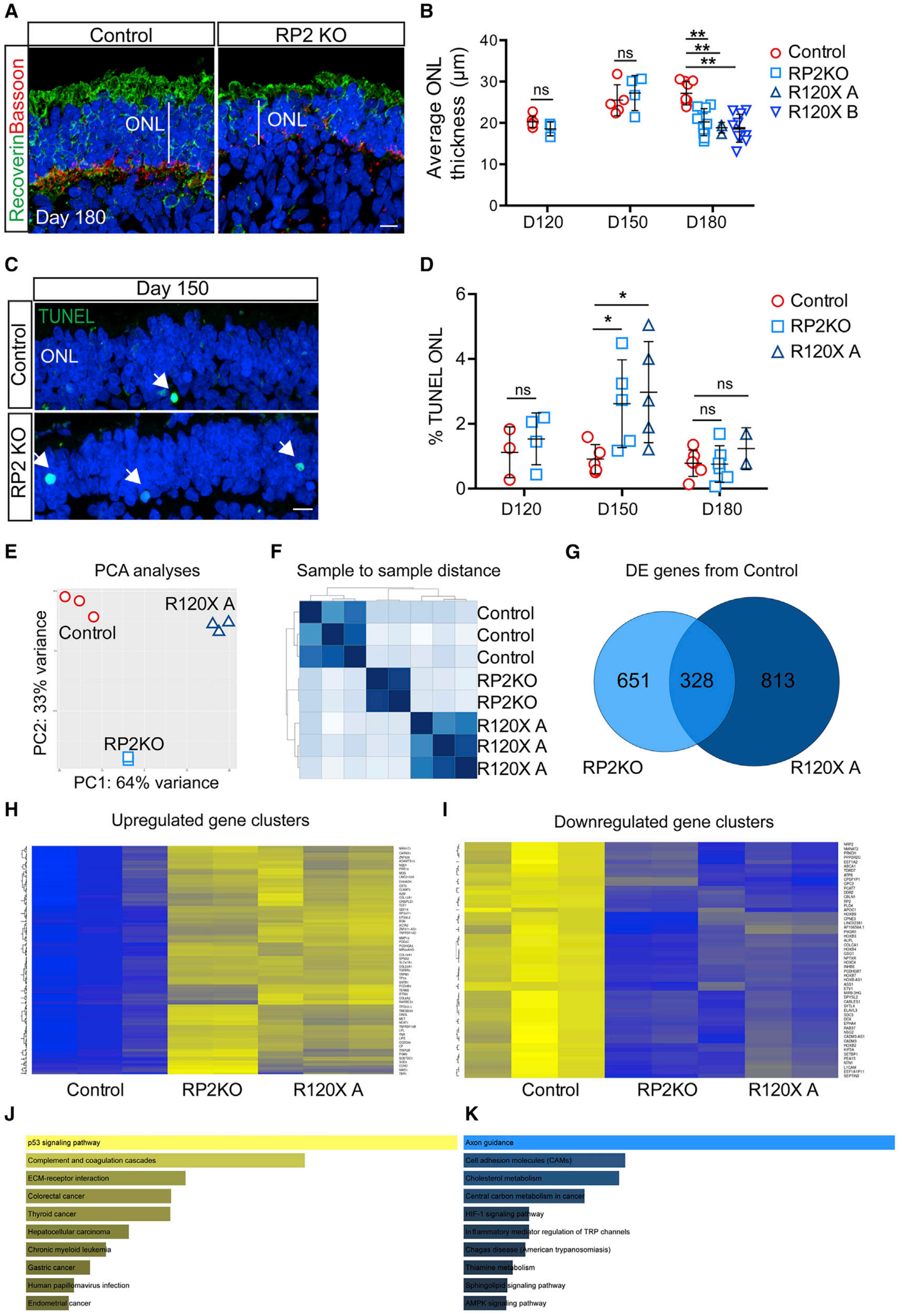

(legend on next page)

D180. Whereas, the RP2 KO ROs had a significantly higher percentage of TUNEL-positive cells in the ONL at D150. There was no significant difference between RP2 KO and controls at D120 or D180, suggesting a peak of cell death during photoreceptor differentiation and maturation around D150 in RP2-deficient cell lines. This was confirmed in R120X-A ROs, with an increase in TUNEL reactivity at D150, which had resolved by D180 (Figure 2D). These data show that there is a peak in photoreceptor cell death that correlates with their maturation and the time course of increased rhodopsin expression.

Gene Expression Changes Associated with Loss of RP2

To investigate gene expression changes that might be associated with the death of photoreceptors in the RP2 null organoids, RNA sequencing (RNA-seq) was performed on D150 controls, RP2 KO, and R120X-A ROs. Principal-component analyses and sample to sample distance shows that the gene expression profiles of the RP2 KO ROs were between the R120X-A patient line and the parental isogenic control (Figures 2E and 2F). There were 328 shared differentially expressed (DE) genes between the RP2 KO and R120X ROs compared with control (Figures 2G–2I). By contrast there were 651 and 813 DE genes between control and RP2 KO and R120X-A ROs, respectively, that were not shared. Kyoto Encyclopedia of Genes and Genomes (KEGG) pathway analyses of the shared DE genes revealed that the "p53 signaling pathway" was the major upregulated pathway, whereas the major downregulated pathway was "axon guidance" (Figures 2J and 2K). KEGG pathway analyses of the axon guidance-related changes showed that expression of genes in pathways that stimulate axon outgrowth, attraction, and repulsion were reduced (Figure S3). Whereas further investigation of the KEGG apoptosis and p53 signaling pathways showed that a number of pro-apoptotic genes, such as p21, BAX, and PUMA, were upregulated in both RP2 null ROs (Figure S3), supporting the observation that loss of RP2 induces cell death in ROs.

Rod Photoreceptor Differentiation and Survival Is Compromised in RP2 KO and R120X ROs

To identify which types of photoreceptor cells were most affected by the loss of RP2, rods and cones were stained with rhodopsin and cone arrestin at D180 (Figure 3A). Strikingly, in the RP2 KO ROs, there was reduced immunoreactivity for rhodopsin compared with isogenic controls. Unlike recoverin expression, which was widespread in the RP2 KO ROs, rhodopsin was restricted to patches of the ONL. To assess photoreceptor gene expression, we quantified rhodopsin (*RHO*) and rod transducin (*GNAT1*) expression by qPCR. In the controls both *RHO* and *GNAT1* showed an age-dependent increase, with a major increase between D150 and D180. In contrast, the RP2 KO ROs had reduced expression of *RHO* and *GNAT1* mRNA at D180, but not D120 or D150, confirming that this difference manifested from D150 onward. The percentage of photoreceptors that were immunoreactive for rhodopsin was quantified as a percentage of DAPI-positive nuclei across the full length of the ONL in sections from all ROs (Figure 3C). The controls showed an increase in rhodopsin-positive cells as the ROs matured. Despite variation between individual ROs, there was a significant difference in the percentage of rhodopsin-expressing cells in mature ROs (D180) between the isogenic control and RP2 KO cell lines (Figures 3A and 3C). Furthermore, the two R120X lines also showed very few rhodopsin-positive photoreceptors at D180, similar to the RP2 KO ROs (Figure 3C). By contrast, the percentage of cone arrestin-positive cells was significantly increased in the RP2 KO and R120X ROs compared with controls at D180, and the mRNA (*ARR3*) was also increased in the RP2 KO ROs at D150 and D180

Figure 2. Photoreceptor Differentiation-Associated Cell Death in RP2 KO Organoids

(A) ICC of control and RP2 KO retinal organoids showing reduced ONL thickness in RP2 KO. Recoverin staining demarcates the ONL terminating in the synaptic layer stained with Bassoon. Scale bar, 10 μm.

(B) Mean ONL thickness per organoid was measured from tilescans of cryosections of a whole organoid at D120, D150 (n = 5 control; n = 4 RP2 KO at both time points), and D180. Significant ONL thinning was recorded at D180 in RP2 KO (n = 9 independent organoids) and R120X lines (n = 3 R120X A organoids, n = 9 R120X B organoids), but not in controls (n = 10 independent organoids; $p \leq 0.01$; mean ± SD).

(C) TUNEL reactive nuclei (arrows) in the ONL of RP2 KO and isogenic control organoids at D150. Scale bar, 10 μm.

(D) Quantification of TUNEL reactivity. RP2 KO organoids had a significantly higher proportion of TUNEL-positive cells at D150 (n = 5 independent organoids $p \leq 0.05$, mean ± SD) but not at D120 (n = 3 control; n = 4 RP2 KO) or D180 (n = 5 control; n = 6 RP2 KO). R120X organoids also had increased TUNEL reactivity at D150 (n = 5 at D150 n = 2 at D180 independent organoids).

(E) Principal-component analyses of RNA-seq data from ROs (n = 3 control and R120X A, n = 2 RP2 KO independent organoids).

(F) Sample to sample distance between samples.

(G) Venn diagram showing differentially expressed genes between RP2 KO and control and R120X and control and common genes.

(H) Heatmap showing upregulated clusters of differentially expressed genes (blue, lower expression; yellow, higher expression).

(I) Heatmap of downregulated clusters of differentially expressed genes.

(J) KEGG pathway analyses of upregulated genes.

(K) KEGG pathway analyses of downregulated genes.

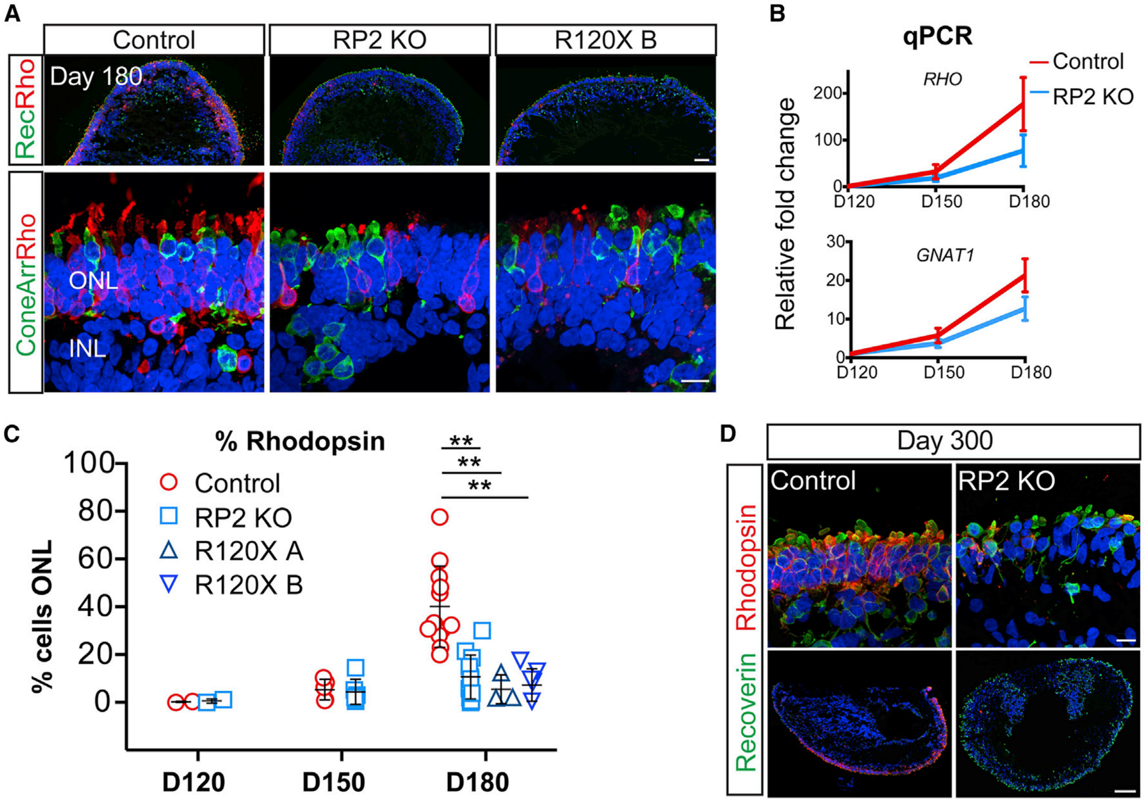

Figure 3. Reduced Number of Rod Cells in RP2 KO and R120X Patient Retinal Organoids

(A) ICC of retinal organoids. Low (upper panel) and high (lower panel) power magnification of recoverin, rhodopsin, and cone arrestin immunoreactivity in the ONL at D180 in control, RP2 KO, and R120X RP2 retinal organoids. Scale bars, 50 μm and 10 μm.

(B) *RHO* and *GNAT1* levels in retinal organoids. qPCR showing relative fold change in mRNA in control and RP2 KO retinal organoids at D120, D150, and D180 (n = 3, 3, 4 independent organoids). Mean ± SEM.

(C) Quantification of rhodopsin-positive cells as a percentage of ONL in control, RP2 KO and R120X patient cell lines from D120 to D180 of differentiation. Each data point represents the mean of 1 independent organoid, counts are from tilescans of whole organoid cross-sections (D120 n = 2 control, n = 2 RP2 KO; D150 n = 5 control, n = 6 RP2 KO; D180 n = 12 control, n = 11 RP2 KO, n = 3 R120X A, n = 5 R120X B; $^{**}p \leq 0.01$; mean ± SD).

(D) High and low magnification of control and RP2 KO organoids at D300 of differentiation stained with recoverin and rhodopsin. Scale bars, 10 μm (upper panel) and 100 μm (lower panel).

(Figure S4), suggesting that the defect primarily affects rods and not cones. There was no significant difference in bipolar cell numbers (Chx10/PKCα) between control and RP2 KO ROs (Figure S4). To exclude that these differences might be attributed to simply a delay in the rate of maturation between the cell lines, control and RP2 KO ROs were maintained in culture for a further 120 days to D300. The RP2 KO had fewer rhodopsin-positive cells relative to the control ROs at D300, whereas recoverin-positive photoreceptors were maintained (Figure 3E).

AAV2/5 Efficiently Transduces ROs to Augment RP2 Expression in RP2 Null Photoreceptors

AAVs are able to transduce post-mitotic rods and cones in mice following subretinal injection (Sarra et al., 2002) and in iPSC-derived rod and cone photoreceptors in ROs with varying efficiency (Gonzalez-Cordero et al., 2018). To assess the ability of AAVs to deliver RP2 to deficient photoreceptors, RP2 KO ROs were transduced with AAV2/5.CAGp.RP2 (Figure 4A) at D140, before the observed onset of ONL thinning, and harvested at D180. At D180, RP2 protein could be detected by ICC throughout the ONL (Figure 4B). At higher magnification, the RP2 signal was detected on the plasma membrane of photoreceptors (Figure 4C). The percentage of RP2-positive cells was determined by scoring nuclei with closely adjacent RP2 signal on the plasma membrane that matched that cell morphology. The transduction efficiency was 90% ± 7% in the ONL (Figure 4D). This included both rhodopsin-positive rod cells

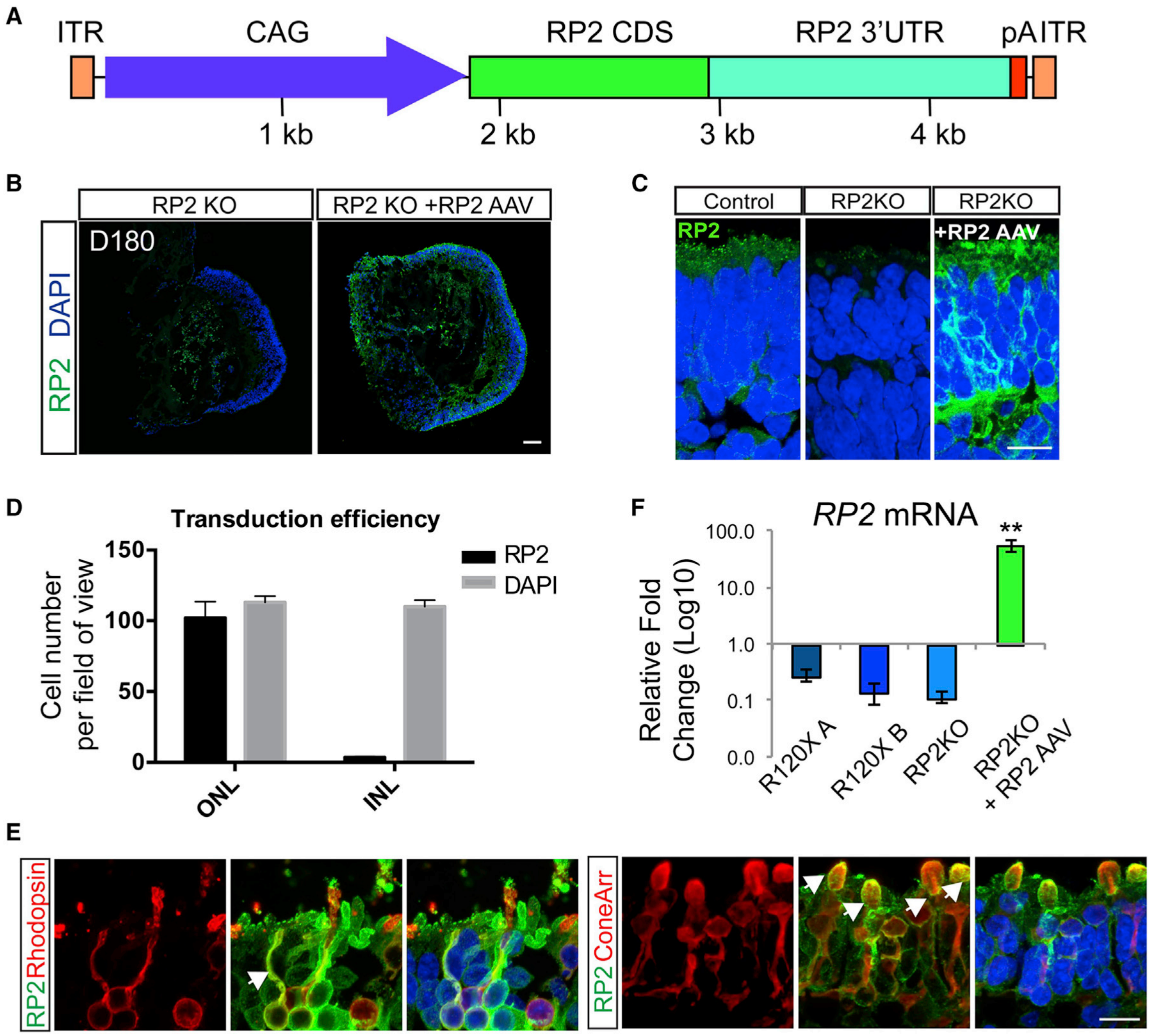

Figure 4. AAV2/5 RP2 Efficiently Transduces Rod and Cone Photoreceptors

(A) Schematic of AAV construction. CAG promoter (blue); RP2 CDS (green); RP2 3′ UTR (teal); pA (red, minimal rabbit b globin poly A); ITR (orange).

(B) RP2 expression in RP2 KO retinal organoids after transduction. ICC on RP2 KO retinal organoid cryosections 6 weeks after transduction with AAV2/5 CAG RP2 showing RP2 expression across the photoreceptor layer (ONL). Scale bar, 50 μm.

(C) High-power magnification of RP2 immunoreactivity. Scale bar, 10 μm.

(D) Cells with RP2 immunoreactivity in the ONL and INL scored against DAPI (mean = 90% ± 7% ONL versus 3% ± 0.4% INL, n = 3 independent organoids; mean ± SD).

(E) ICC co-staining RP2 with cone arrestin or rhodopsin showing AAV-driven RP2 expression in both rod and cone photoreceptors. Scale bar, 10 μm.

(F) qPCR of RP2 mRNA transcript levels in AAV-transduced RP2 KO organoids relative to endogenous expression in control organoids (n = 3 independent organoids; mean = 55-fold ±7.7 SEM).

(Figure 4E) and cone arrestin-positive cone cells (Figure 4E). Sporadic RP2 staining in the inner retinal layers was also visible, suggesting that a small proportion of the AAV is able to penetrate the RO and transduce inner retinal cells (Figure S5). Analysis of mRNA levels by qPCR revealed an average 55-fold increase in RP2 transcript relative to control organoids (Figure 4F).

AAV2/5-Driven RP2 Gene Augmentation Improves Photoreceptor Survival

To investigate if the increase in RP2 levels was altering the degenerative phenotype of the ROs, ONL thickness and rhodopsin immunoreactivity were compared in RP2 AAV-transduced versus untransduced RP2 KO ROs at D180 (Figures 5A and 5C). The ONL was stained with

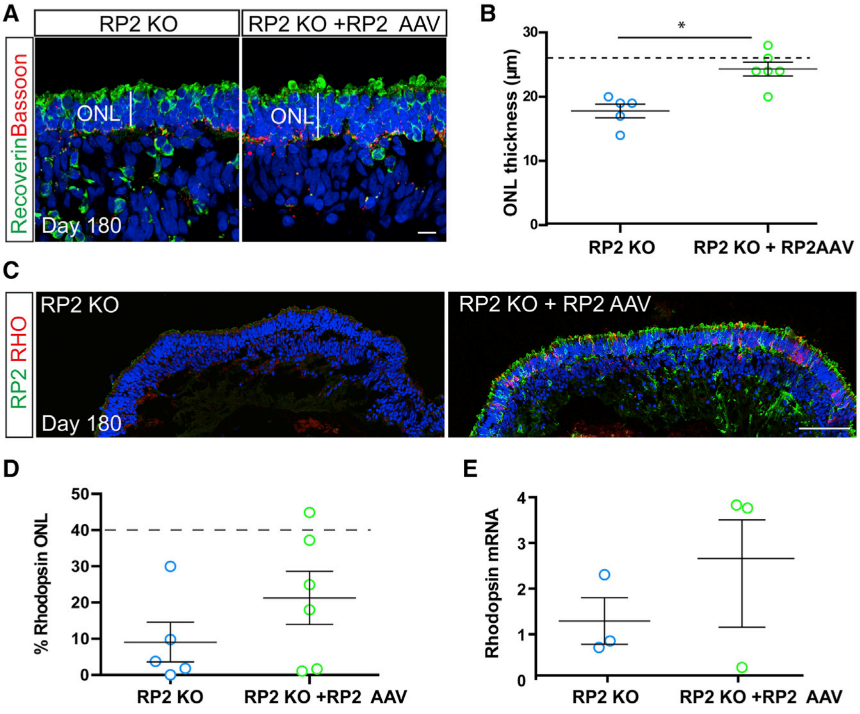

Figure 5. AAV2/5-Driven RP2 Overexpression Rescues Photoreceptor Survival

(A) ICC of retinal organoid ONL with recoverin (green) and Bassoon (red) in RP2 KO control and AAV RP2-treated retinal organoids showing improved ONL thickness in the AAV-treated group. Scale bar, 10 μm.

(B) Quantification of ONL thickness in control and AAV transduced retinal organoids (n = 5 RP2-KO, 6 RP2 KO + AAV independent organoids, *p = 0.016, mean ± SEM). Dotted line represents mean thickness in isogenic control parent cell line.

(C) ICC showing RP2 (green) and rhodopsin (red) expression after transduction. Scale bar, 50 μm.

(D) Quantification of ICC to assess rhodopsin-positive cells in control and RP2 AAV-transduced RP2 KO retinal organoids. Dotted line represents average control values (n = 5 RP2 KO, n = 6 RP2 KO + RP2 AAV independent organoids, p = 0.23; mean ± SEM).

(E) qPCR for *RHO* mRNA levels in control and RP2 AAV-treated RP2 KO retinal organoids (n = 3 independent organoids). Mean ± SEM.

recoverin and Bassoon (Figure 5A). Measurement of the ONL revealed that AAV-RP2-transduced RP2 KO organoids had a significantly thicker ONL than non-transduced controls at D180 ($p \leq 0.01$, Figure 5B) with increased numbers of photoreceptors (Figure S5). The ONL thickness post-transduction was similar to that observed for the isogenic control cells at D180, suggesting near complete rescue. In most, but not all, transduced organoids the percentage of rhodopsin-positive cells was above the average for non-transduced RP2 KOs suggesting that AAV RP2 expression could restore rhodopsin immunoreactivity (Figure 5D). This was also observed at the mRNA level, with up to a 3-fold increase in *RHO* level in transduced ROs; however, one transduced RO showed no response at the level of rhodopsin expression (Figure 5E). The percentage of cone arrestin-positive cells was also reduced following AAV transduction, but *ARR3* mRNA was unchanged (Figure S5).

DISCUSSION

Here, we describe an *in vitro* model of RP2 XLRP and show that AAV gene augmentation of RP2 can successfully reverse measurable and clinically relevant disease phenotypes. The combination of iPSC reprogramming, CRISPR gene-editing technology, and AAV gene delivery has enabled a side by side comparison of RP2 KO, isogenic controls, and XLRP patient ROs, to obviate some of the inherent variation in iPSC-derived organoid models.

Differentiation of iPSCs presents an opportunity to probe genetic disease mechanisms in the cells and tissues that are affected by specific genetic changes. This is particularly useful in the case of ubiquitously expressed genes, such as *RP2*, that have disease pathology restricted to a specific tissue, such as the retina. Here, we used iPSC reprogramming technology in combination with CRISPR/Cas9 gene editing to probe disease mechanisms in *RP2* XLRP.

All of the RP2-deficient cell lines used in this study successfully developed 3D ROs with advanced morphological features, including lamination and formation of rod and cone photoreceptors. Strikingly, we observed a significant decline in photoreceptor cell numbers, specifically rhodopsin-positive rods, and significantly thinner ONL after 150 days of differentiation in all RP2-deficient lines. Widespread rhodopsin expression is one of the later events in RO development, in keeping with the developmental time line *in vivo*; however, the presence of other late-emerging cell markers in the RP2 KO and patient cell lines, such as cone arrestin-positive cone photoreceptors, PKCα-positive bipolar cells, and OS structures, together with the persistence of this rhodopsin-deficient phenotype up to 300 days of differentiation suggests that this is not merely a case of developmental delay.

This phenotype has not been reported in any existing animal models of RP2 XLRP (Li et al., 2013; Zhang et al., 2015),

suggesting that this is either specific to human retina, or to the *in vitro* organoid system. Despite the relative stability of ROs in comparison with *ex vivo* retinal explant culture, which can be maintained for around 14 days (Johnson and Martin, 2008), it is likely that the conditions for retinal cell maintenance in culture are suboptimal. The absence of an opposing RPE monolayer, nutrient and oxygen deprivation through the lack of vascular blood supply, or the lack of connectivity for the inner retinal neurons, for example, may be sources of stress that accelerate the onset of disease phenotypes. This may go some way to explain the early "age" of detectable changes relative to mouse and human RP2 XLRP *in vivo*. In RP2 KO mice, ONL thinning is detectable only at 5 months of age in one model (Li et al., 2013), whereas almost no ONL thinning was observed at 12 months of age in another model (Zhang et al., 2015). Here, we detected measurable differences in photoreceptor survival at the onset of rod differentiation and rhodopsin expression. Cell death is an important, non-pathological process during retinal development (Vecino et al., 2004), but is increased during differentiation in the photoreceptor cell layer of RP2-deficient ROs, linking rod maturation temporally with rod cell death in this model. Interestingly, ROs from an individual with a mutation in *RPGR*, the other major form of XLRP, also showed signs of photoreceptor cell death at around 150 days of RO differentiation, which was rescued by CRISPR-mediated repair of the RPGR mutation in the iPSC (Deng et al., 2018). The induction of p53 pathways was also observed in the RPGR retinal organoid model (Deng et al., 2018), suggesting that this could be a common pathway in both forms of XLRP in human ROs. Therefore, photoreceptor cell death appears to be a feature in ROs for some forms of inherited retinal disease, but the precise mechanisms and whether there is an opportunity to inhibit the cell death process will require further investigation.

AAV is a highly efficient method of delivering therapeutic genes to photoreceptor cells and AAV2/5 has been shown to effectively transduce photoreceptors in a variety of species *inter alia* mice (Palfi et al., 2012), non-human primates (Boye et al., 2012), and human retinal explants (Wiley et al., 2018). The use of AAV in human ROs is currently limited, but studies have reported varying efficiency of transduction, which could be attributed to age of treatment, time of harvesting, vector tropisms, viral titer, the promotor used, and the sensitivity of detection of the transgene, e.g., antibody versus intrinsic fluorescence of a reporter (Garita-Hernandez et al., 2020; Gonzalez-Cordero et al., 2018; Quinn et al., 2019). Here, we demonstrate highly efficient transduction using an AAV2/5 vector with RP2 under the control of a CAG promoter. The AAV appears to deliver RP2 more or less exclusively to the photoreceptor cells despite the use of a ubiquitous promotor. This may be due to a combination of vector tropisms and the organization of the organoid, where the photoreceptor layer is outermost and, therefore, in contact the viral particles in solution. In addition, the OLM may prevent diffusion of AAV into the inner retinal layers. The interaction of AAV and photoreceptors in this system is analogous to the situation following subretinal delivery. Indeed, in a human treatment context, the optimal route of administration for this vector would be subretinal injection. Although intravitreal injection is less invasive, most AAV serotypes cannot transduce photoreceptors efficiently when administered intravitreally.

AAV RP2 was delivered at D140, before the onset of the increase in photoreceptor TUNEL reactivity and ONL thinning, but late enough to ensure efficient uptake, following reports of inefficient transduction at earlier time points (Gonzalez-Cordero et al., 2018). Not only was RP2 efficiently expressed at the mRNA and protein level, but it was also able to rescue the ONL-thinning phenotype in RP2 KO ROs implying a protective effect of RP2 overexpression in photoreceptor cells. Interestingly, although RP2 was expressed at over 55-fold the endogenous level, we did not observe any overt deleterious effects. In a previous study, some toxicity was observed with RP2 overexpression in mice 3 months after administration of the highest dose of AAV-RP2 vector (1×10^9 viral genomes [vg]/eye), although the level of RP2 overexpression in transduced mouse eyes was not defined (Mookherjee et al., 2015). As RP2 is a GAP for ARL3, it is possible that increasing RP2 to a high level would inhibit the production of ARL3-GTP and mimic the effect of loss of the ARL3 GEF, ARL13b, which causes Joubert syndrome; however, this might not occur in the cellular context because of the spatial separation of the proteins, with RP2 predominantly at the base of the cilium and ARL13b in the axoneme, which leads to a gradient of ARL3-GTP in the cilium. Despite very high levels of RP2, once ARL3 is trafficked into the cilium it can be converted to ARL3-GTP by ARL13b; however, this would not exclude other activities of overexpressed RP2 from disrupting homeostasis. Therefore, additional studies evaluating the therapeutic index for AAV-RP2 therapies in ROs, and/or the primate eye, would be of value, before translation to the clinic.

Although RP2 is expressed in multiple retinal cell types, it remains to be established whether optimal rescue of the RP2 disease phenotype may require expression of a replacement *RP2* gene in other retinal cell types, such as the RPE. The use of a ubiquitous promoter in the current study should, in principle, enable expression of the therapeutic gene in multiple cell types. Additional studies will be required to fully elucidate the requirement, or otherwise, for RP2 in the RPE, to develop optimal RP2 gene therapies. In contrast to the current study, Mookherjee et al. (2015) used the photoreceptor-specific rhodopsin kinase

promoter to drive expression of the RP2 gene and achieved a partial rescue of the cone phenotype in a null RP2 mouse model but had no beneficial effect on the rod phenotype.

This study provides insights into the use of ROs for retinal disease modeling, with a phenotype related to loss of RP2 that is unique to the human retina cell culture model. Importantly, the photoreceptor cell death in the RP2-deficient organoids correlates with the timing of rod cell maturation and rhodopsin expression. Furthermore, we highlight how the use of AAV for the restoration of RP2 expression can reverse this phenotype, and as such could be further investigated as a potential therapeutic avenue for the treatment of XLRP.

EXPERIMENTAL PROCEDURES

Reprogramming and Gene Editing

Following informed consent, a skin biopsy was obtained from study participants to obtain dermal fibroblasts. The study followed the tenets of the Declaration of Helsinki and was approved by the Moorfields Eye Hospital and Royal Victoria Eye and Ear Hospital Dublin Research Ethics Committees. iPSCs were generated from two unrelated R120X individuals and control fibroblasts (BJ fibroblast ATCC CRL-2522) as described previously (Schwarz et al., 2015). RP2 KO iPSCs were produced by simultaneous reprogramming and gene editing using a method described previously (Howden et al., 2015). Guide RNAs were designed to target exon 2 of *RP2* (see Table S1 for sequences), and were cloned into the pSpCas9(BB)-2A-Puro (PX459) V2.0 plasmid (Addgene plasmid no. 62,988) according to a previously described protocol (Ran et al., 2013). iPSC clones were manually isolated, genomic DNA extracted (Promega), and PCR amplified with primers designed around the target site (Table S1). iPSC clones were analyzed by Sanger sequencing to confirm *RP2* gene disruption. Off-Spotter (https://cm.jefferson.edu/Off-Spotter/) was used to predict off-targets for the selected gRNA and the top 10 off-targets were assessed with Sanger sequencing, which detected no changes (Figure S1D; Table S1). In addition, all DE genes from the RNA-seq analysis were cross-referenced with off-target predictions and a further eight potential off-target sites with four or five mismatches were analyzed (Figure S1E; Table S1). These showed no sequence changes.

Differentiation of iPSCs to ROs

RO differentiation was carried out as described previously (Nakano et al., 2012; Zhong et al., 2014). In brief, iPSCs were grown to near confluence in E8 medium before detaching colonies in gentle dissociation buffer (STEMCELL Technologies) to form embryoid bodies (EBs). EBs were transitioned to neural induction medium in the presence of blebbistatin, before plating down at a density of approximately 20 EBs per cm^2. Emerging transparent pouches of neuroepithelium were isolated using a needle and cultured in suspension in retinal maturation media +0.5 μm retinoic acid up to D140, after which retinoic acid was removed. Alternatively EBs were generated by single-cell dissociation and forced aggregation in 96-well V-bottomed plates and cultured in suspension thereafter (Nakano et al., 2012). Days of culture are ±3days.

AAV Production

The RP2 replacement vector comprising a CAG promoter, human RP2 CDS, RP2 3′ UTR, including an RP2 poly(A) sequence, and a minimal rabbit β-globin poly(A), was synthesized by GeneArt (Life Technologies) and cloned into pAAV-MCS using flanking NotI sites. More detail of sequences is in the Supplemental Information. Recombinant AAV2/5 viruses were generated by helper virus-free, triple transfection (Xiao et al., 1998). Human embryonic kidney cells (accession number CRL-1573; ATCC, USA) were transfected with pAAV-RP2, pRep/Cap5 (Hildinger et al., 2001) and pHelper (Agilent Technologies, USA) at a ratio of 1:1:2, as described previously (O’reilly et al., 2007). Seventy-two hours after transfection, AAV particles were purified from the clarified lysate by differential precipitation with polyethylene glycol followed by cesium gradient centrifugation (Ayuso et al., 2010). AAV-containing fractions were dialyzed against PBS supplemented with Pluronic F68 (0.001%; Bennicelli et al., 2008). Genomic titers (vg/mL) were determined by quantitative real-time PCR (qPCR; Rohr et al., 2002).

AAV Treatment

RP2 AAV was prepared to a final titer 4.73×10^{12} vg/mL. At 140 days, organoids with brush borders visible by light microscopy, which show inner segment and OS development, were transferred to the well of 96-well plate and incubated with 1E11 viral genomes in 75 μL media for 8 h before topping the media up to 200 μL. Followed by 50/50 medium changes every 2 days until day 180.

RNA Extraction and qPCR

ROs or half ROs were subjected to RNA extraction using an RNeasy MicroKit (QIAGEN) and cDNA synthesis was performed using a Tetro cDNA synthesis kit (Bioline). qPCR was carried out on an Applied Biosystems 7900HT Fast Real-Time PCR system using the SYBR Green method using 1 μL cDNA per triplicate. Data from the qPCR were normalized to the geometric mean of the expression of two internal reference genes in each sample. *POLR2A* and *MAN1B1* were chosen due to their consistency across the sample groups, which was determined using the GeNorm algorithm (Vandesompele et al., 2002). Primers were designed to cross exon boundaries (see Table S2).

RNA-Seq

Three ROs from BJ controls and R120X cell lines and two ROs from the RP2 KO cell line were harvested at D150. The RNA was extracted with the RNeasy micro kit (QIAGEN) following the manufacturer’s instructions, followed by paired-end sequencing at 100 million read depth for each sample (Illumina, Otogenetics, Atlanta, GA, USA). Raw.fastq sequences were cleaned from any residual sequencing adapter using cutadapt with parameters -m 20 and -e 0.1. Fragments were then aligned to the human genome

(build 38, Ensembl version 92) using STAR and genes counted using featurecounts.

Differential expression analysis was then performed using the DESeq2 pipeline. Initial inspection of the dataset showed biases, which were blind estimated and corrected using the sva package (DESeq2 manual). The differential expression analysis showed that 1,141 genes were differentially expressed between the R120X ROs and the control, whereas 979 genes were differentially expressed between the RP2 KO ROs and the isogenic control and 328 genes were common to the 2 RP2-depleted backgrounds. Of these, 232 genes were consistently upregulated and 56 were downregulated. The list of DE genes is shown in Table S3. Finally, we used the web platform Enricher to source any significant pathway (https://amp.pharm.mssm.edu/Enrichr/). According to the KEGG there was a significant upregulation of the "p53 signaling pathway" in the RP2 KO and RP2-R120X retina (Figure S3).

Immunofluorescence

ROs were either fixed whole or bisected under a dissecting microscope using micro scissors (Fine Science Tools) with the other half processed for RNA extraction. ROs or half ROs were fixed in 4% paraformaldehyde at 4°C for 20–30 min before cryoprotection by immersion overnight in 30% sucrose/PBS. Following orientation under a dissecting microscope in OCT they were frozen and cryosectioned into 6-μm sections. For ICC, slides were blocked in 10% donkey serum, 0.1% Triton X for 1 h before incubation with primary antibodies for 2 h (see Table S4 for primary antibodies) and donkey anti-rabbit or mouse Alexa Flour 488 or 555 secondary antibodies for 1 h (Thermo Fisher). Nuclei were visualized using DAPI (2 μg/mL) staining for all images.

Imaging

All images were obtained using a Carl Zeiss LSM700 or LSM 710 laser scanning confocal microscope. Images were exported from Zen imaging software and prepared using Adobe Photoshop and Illustrator CS4. All measurements were performed in Fiji (Schindelin et al., 2012). For quantification of TUNEL, rod and cone cell numbers, and ONL thickness measurements, the tilescan function was used to obtain images of whole organoid sections at 40× magnification (more detail in Supplemental Information). Individual rod-, cone-, and TUNEL-positive cell numbers in the ONL were manually counted across the whole section. ONL thickness was calculated by measuring the length and area of the whole recoverin-positive ONL in the DAPI channel using Fiji.

Electron Microscopy

ROs were fixed overnight in 3% glutaraldehyde and 1% paraformaldehyde buffered to pH 7.4 with 0.08 M sodium cacodylate-HCl buffer. After rinsing in 0.1 M sodium cacodylate-HCl buffer (pH 7.4) twice for 5 min, the ROs were post-fixed in 1% aqueous osmium tetroxide for 2 h, dehydrated by passage through ascending alcohols (10-min steps, 1× 50%–90% and 3× 100%) and two changes of propylene oxide, and infiltrated overnight with a 1:1 mixture of propylene oxide:araldite on a rotator. Finally, ROs were infiltrated with araldite resin over 4–6 h and embedded in fresh resin, which was then cured by overnight incubation at 60°C. Semithin sections (0.75 μm) were stained with a 1% mixture of toluidine blue-borax in 50% ethanol, and ultrathin sections were contrasted with Reynolds lead citrate for imaging in a JEOL 1010 TEM operating at 80 kV. Images were captured using a Gatan Orius CCD camera using Digital Micrograph software.

Statistical Analysis

Statistical analysis was carried out on GraphPad Prism. Data were subjected to two-way ANOVA and/or by multiple t tests using the Holm-Sidak method, with $\alpha = 5.00\%$. Each time point was analyzed individually, without assuming a consistent standard deviation (SD). Significance was determined at a p value of < 0.05.

Data and Code Availability

The RNA-seq data are available at GEO: GSE148300.

SUPPLEMENTAL INFORMATION

Supplemental Information can be found online at https://doi.org/10.1016/j.stemcr.2020.05.007.

AUTHOR CONTRIBUTIONS

A.L., K.J., C.S., D.O., A.B.P., N.S., R.G., M.J.H., N.E.S., A.P., and N.C. performed the experiments and/or analyzed the data. A.L., K.J., C.S., D.O., N.E.S., G.J.F., A.J.H., and M.E.C. conceived the hypothesis and designed the experiments. A.L., K.J., C.S., G.J.F., A.J.H., and M.E.C. drafted the manuscript. All authors edited the draft manuscript.

ACKNOWLEDGMENTS

We are grateful to Robert Molday (UBC), Cheryl Craft (USC), Peter MacLeish (Morehouse School of Medicine), and Wolfgang Baehr (University of Utah) for providing antibodies. This work was funded by Moorfields Eye Charity through a generous donation (to A.J.H. and M.E.C.), Fight for Sight (to M.E.C. and A.J.H.), the NC3Rs (to M.E.C.), and the Wellcome Trust (to M.E.C.). It was also supported by the NIH Research Biomedical Research Centre at Moorfields Eye Hospital NHS Foundation Trust and UCL Institute of Ophthalmology (A.J.H. is NIHR BRC Faculty). Support was also provided by the Health Research Board of Ireland (HRB), Fighting Blindness Ireland (FBI), the Medical Research Charities Group (MRCG), and Science Foundation Ireland (to G.J.F.).

Received: February 7, 2020
Revised: May 13, 2020
Accepted: May 13, 2020
Published: June 11, 2020

REFERENCES

Ayuso, E., Mingozzi, F., Montane, J., Leon, X., Anguela, X.M., Haurigot, V., Edmonson, S.A., Africa, L., Zhou, S., High, K.A., et al. (2010). High AAV vector purity results in serotype- and tissue-independent enhancement of transduction efficiency. Gene Ther. *17*, 503–510.

Bennicelli, J., Wright, J.F., Komaromy, A., Jacobs, J.B., Hauck, B., Zelenaia, O., Mingozzi, F., Hui, D., Chung, D., Rex, T.S., et al. (2008). Reversal of blindness in animal models of leber congenital amaurosis using optimized AAV2-mediated gene transfer. Mol. Ther. *16*, 458–465.

Boye, S.E., Alexander, J.J., Boye, S.L., Witherspoon, C.D., Sandefer, K.J., Conlon, T.J., Erger, K., Sun, J., Ryals, R., Chiodo, V.A., et al. (2012). The human rhodopsin kinase promoter in an AAV5 vector confers rod- and cone-specific expression in the primate retina. Hum. Gene Ther. *23*, 1101–1115.

Breuer, D.K., Yashar, B.M., Filippova, E., Hiriyanna, S., Lyons, R.H., Mears, A.J., Asaye, B., Acar, C., Vervoort, R., Wright, A.F., et al. (2002). A comprehensive mutation analysis of RP2 and RPGR in a North American cohort of families with X-linked retinitis pigmentosa. Am. J. Hum. Genet. *70*, 1545–1554.

Capowski, E.E., Samimi, K., Mayerl, S.J., Phillips, M.J., Pinilla, I., Howden, S.E., Saha, J., Jansen, A.D., Edwards, K.L., Jager, L.D., et al. (2019). Reproducibility and staging of 3D human retinal organoids across multiple pluripotent stem cell lines. Development *146*.

Deng, W.L., Gao, M.L., Lei, X.L., Lv, J.N., Zhao, H., He, K.W., Xia, X.X., Li, L.Y., Chen, Y.C., Li, Y.P., et al. (2018). Gene correction reverses ciliopathy and photoreceptor loss in iPSC-derived retinal organoids from retinitis pigmentosa patients. Stem Cell Reports *10*, 1267–1281.

Evans, R.J., Schwarz, N., Nagel-Wolfrum, K., Wolfrum, U., Hardcastle, A.J., and Cheetham, M.E. (2010). The retinitis pigmentosa protein RP2 links pericentriolar vesicle transport between the Golgi and the primary cilium. Hum. Mol. Genet. *19*, 1358–1367.

Gagliardi, G., Ben M'barek, K., and Goureau, O. (2019). Photoreceptor cell replacement in macular degeneration and retinitis pigmentosa: a pluripotent stem cell-based approach. Prog. Retin. Eye Res. *71*, 1–25.

Garita-Hernandez, M., Routet, F., Guibbal, L., Khabou, H., Toualbi, L., Riancho, L., Reichman, S., Duebel, J., Sahel, J.A., Goureau, O., and Dalkara, D. (2020). AAV-mediated gene delivery to 3D retinal organoids derived from human induced pluripotent stem cells. Int. J. Mol. Sci. *21*.

Gonzalez-Cordero, A., Goh, D., Kruczek, K., Naeem, A., Fernando, M., Kleine Holthaus, S.M., Takaaki, M., Blackford, S.J.I., Kloc, M., Agundez, L., et al. (2018). Assessment of AAV vector tropisms for mouse and human pluripotent stem cell-derived RPE and photoreceptor cells. Hum. Gene Ther. *29*, 1124–1139.

Gonzalez-Cordero, A., Kruczek, K., Naeem, A., Fernando, M., Kloc, M., Ribeiro, J., Goh, D., Duran, Y., Blackford, S.J.I., Abelleira-Hervas, L., et al. (2017). Recapitulation of human retinal development from human pluripotent stem cells generates transplantable populations of cone photoreceptors. Stem Cell Reports *9*, 820–837.

Gotthardt, K., Lokaj, M., Koerner, C., Falk, N., Giessl, A., and Wittinghofer, A. (2015). A G-protein activation cascade from Arl13B to Arl3 and implications for ciliary targeting of lipidated proteins. eLife *4*.

Grayson, C., Bartolini, F., Chapple, J.P., Willison, K.R., Bhamidipati, A., Lewis, S.A., Luthert, P.J., Hardcastle, A.J., Cowan, N.J., and Cheetham, M.E. (2002). Localization in the human retina of the X-linked retinitis pigmentosa protein RP2, its homologue cofactor C and the RP2 interacting protein Arl3. Hum. Mol. Genet. *11*, 3065–3074.

Hardcastle, A.J., Thiselton, D.L., Van Maldergem, L., Saha, B.K., Jay, M., Plant, C., Taylor, R., Bird, A.C., and Bhattacharya, S. (1999). Mutations in the RP2 gene cause disease in 10% of families with familial X-linked retinitis pigmentosa assessed in this study. Am. J. Hum. Genet. *64*, 1210–1215.

Hildinger, M., Auricchio, A., Gao, G., Wang, L., Chirmule, N., and Wilson, J.M. (2001). Hybrid vectors based on adeno-associated virus serotypes 2 and 5 for muscle-directed gene transfer. J. Virol. *75*, 6199–6203.

Howden, S.E., Maufort, J.P., Duffin, B.M., Elefanty, A.G., Stanley, E.G., and Thomson, J.A. (2015). Simultaneous reprogramming and gene correction of patient fibroblasts. Stem Cell Reports *5*, 1109–1118.

Ismail, S.A., Chen, Y.X., Rusinova, A., Chandra, A., Bierbaum, M., Gremer, L., Triola, G., Waldmann, H., Bastiaens, P.I., and Wittinghofer, A. (2011). Arl2-GTP and Arl3-GTP regulate a GDI-like transport system for farnesylated cargo. Nat. Chem. Biol. *7*, 942–949.

Jayasundera, T., Branham, K.E., Othman, M., Rhoades, W.R., Karoukis, A.J., Khanna, H., Swaroop, A., and Heckenlively, J.R. (2010). RP2 phenotype and pathogenetic correlations in X-linked retinitis pigmentosa. Arch. Ophthalmol. *128*, 915–923.

Johnson, T.V., and Martin, K.R. (2008). Development and characterization of an adult retinal explant organotypic tissue culture system as an in vitro intraocular stem cell transplantation model. Invest. Ophthalmol. Vis. Sci. *49*, 3503–3512.

Li, L., Khan, N., Hurd, T., Ghosh, A.K., Cheng, C., Molday, R., Heckenlively, J.R., Swaroop, A., and Khanna, H. (2013). Ablation of the X-linked retinitis pigmentosa 2 (Rp2) gene in mice results in opsin mislocalization and photoreceptor degeneration. Invest. Ophthalmol. Vis. Sci. *54*, 4503–4511.

Mookherjee, S., Hiriyanna, S., Kaneshiro, K., Li, L., Li, Y., Li, W., Qian, H., Li, T., Khanna, H., Colosi, P., et al. (2015). Long-term rescue of cone photoreceptor degeneration in retinitis pigmentosa 2 (RP2)-knockout mice by gene replacement therapy. Hum. Mol. Genet. *24*, 6446–6458.

Nakano, T., Ando, S., Takata, N., Kawada, M., Muguruma, K., Sekiguchi, K., Saito, K., Yonemura, S., Eiraku, M., and Sasai, Y. (2012). Self-formation of optic cups and storable stratified neural retina from human ESCs. Cell Stem Cell *10*, 771–785.

O'reilly, M., Palfi, A., Chadderton, N., Millington-Ward, S., Ader, M., Cronin, T., Tuohy, T., Auricchio, A., Hildinger, M., Tivnan, A., et al. (2007). RNA interference-mediated suppression and replacement of human rhodopsin in vivo. Am. J. Hum. Genet. *81*, 127–135.

Okita, K., Matsumura, Y., Sato, Y., Okada, A., Morizane, A., Okamoto, S., Hong, H., Nakagawa, M., Tanabe, K., Tezuka, K., et al. (2011). A more efficient method to generate integration-free human iPS cells. Nat. Methods *8*, 409–412.

Palfi, A., Chadderton, N., Mckee, A.G., Blanco Fernandez, A., Humphries, P., Kenna, P.F., and Farrar, G.J. (2012). Efficacy of

codelivery of dual AAV2/5 vectors in the murine retina and hippocampus. Hum. Gene Ther. *23*, 847–858.

Parfitt, D.A., Lane, A., Ramsden, C.M., Carr, A.J., Munro, P.M., Jovanovic, K., Schwarz, N., Kanuga, N., Muthiah, M.N., Hull, S., et al. (2016). Identification and correction of mechanisms underlying inherited blindness in human iPSC-derived optic cups. Cell Stem Cell *18*, 769–781.

Quinn, P.M., Buck, T.M., Mulder, A.A., Ohonin, C., Alves, C.H., Vos, R.M., Bialecka, M., Van Herwaarden, T., Van Dijk, E.H.C., Talib, M., et al. (2019). Human iPSC-derived retinas recapitulate the fetal CRB1 CRB2 complex formation and demonstrate that photoreceptors and muller glia are targets of AAV5. Stem Cell Reports *12*, 906–919.

Ran, F.A., Hsu, P.D., Wright, J., Agarwala, V., Scott, D.A., and Zhang, F. (2013). Genome engineering using the CRISPR-Cas9 system. Nat. Protoc. *8*, 2281–2308.

Rohr, U.P., Wulf, M.A., Stahn, S., Steidl, U., Haas, R., and Kronenwett, R. (2002). Fast and reliable titration of recombinant adeno-associated virus type-2 using quantitative real-time PCR. J. Virol. Methods *106*, 81–88.

Russell, S., Bennett, J., Wellman, J.A., Chung, D.C., Yu, Z.F., Tillman, A., Wittes, J., Pappas, J., Elci, O., Mccague, S., et al. (2017). Efficacy and safety of voretigene neparvovec (AAV2-hRPE65v2) in patients with RPE65-mediated inherited retinal dystrophy: a randomised, controlled, open-label, phase 3 trial. Lancet *390*, 849–860.

Sarra, G.M., Stephens, C., Schlichtenbrede, F.C., Bainbridge, J.W., Thrasher, A.J., Luthert, P.J., and Ali, R.R. (2002). Kinetics of transgene expression in mouse retina following sub-retinal injection of recombinant adeno-associated virus. Vis. Res *42*, 541–549.

Schwarz, N., Carr, A.J., Lane, A., Moeller, F., Chen, L.L., Aguila, M., Nommiste, B., Muthiah, M.N., Kanuga, N., Wolfrum, U., et al. (2015). Translational read-through of the RP2 Arg120stop mutation in patient iPSC-derived retinal pigment epithelium cells. Hum. Mol. Genet. *24*, 972–986.

Schwarz, N., Hardcastle, A.J., and Cheetham, M.E. (2012). The role of the X-linked retinitis pigmentosa protein RP2 in vesicle traffic and cilia function. Adv. Exp. Med. Biol. *723*, 527–532.

Schwarz, N., Lane, A., Jovanovic, K., Parfitt, D.A., Aguila, M., Thompson, C.L., da Cruz, L., Coffey, P.J., Chapple, J.P., Hardcastle, A.J., and Cheetham, M.E. (2017). Arl3 and RP2 regulate the trafficking of ciliary tip kinesins. Hum. Mol. Genet. *26*, 2480–2492.

Sharma, T.P., Wiley, L.A., Whitmore, S.S., Anfinson, K.R., Cranston, C.M., Oppedal, D.J., Daggett, H.T., Mullins, R.F., Tucker, B.A., and Stone, E.M. (2017). Patient-specific induced pluripotent stem cells to evaluate the pathophysiology of TRNT1-associated retinitis pigmentosa. Stem Cell Res. *21*, 58–70.

Schindelin, J., Arganda-Carreras, I., Frise, E., Kaynig, V., Longair, M., Pietzsch, T., Preibisch, S., Rueden, C., Saalfeld, S., Schmid, B., et al. (2012). Fiji: an open-source platform for biological-image analysis. Nat. Methods *9*, 676–682.

Shirai, H., Mandai, M., Matsushita, K., Kuwahara, A., Yonemura, S., Nakano, T., Assawachananont, J., Kimura, T., Saito, K., Terasaki, H., et al. (2016). Transplantation of human embryonic stem cell-derived retinal tissue in two primate models of retinal degeneration. Proc. Natl. Acad. Sci. U S A *113*, E81–E90.

Takahashi, K., Tanabe, K., Ohnuki, M., Narita, M., Ichisaka, T., Tomoda, K., and Yamanaka, S. (2007). Induction of pluripotent stem cells from adult human fibroblasts by defined factors. Cell *131*, 861–872.

Vandesompele, J., De Preter, K., Pattyn, F., Poppe, B., Van Roy, N., De Paepe, A., and Speleman, F. (2002). Accurate normalization of real-time quantitative RT-PCR data by geometric averaging of multiple internal control genes. Genome Biol. *3*, research0034.1.

Vecino, E., Hernandez, M., and Garcia, M. (2004). Cell death in the developing vertebrate retina. Int. J. Dev. Biol. *48*, 965–974.

Veltel, S., Gasper, R., Eisenacher, E., and Wittinghofer, A. (2008). The retinitis pigmentosa 2 gene product is a GTPase-activating protein for Arf-like 3. Nat. Struct. Mol. Biol. *15*, 373–380.

Wiley, L.A., Burnight, E.R., Kaalberg, E.E., Jiao, C., Riker, M.J., Halder, J.A., Luse, M.A., Han, I.C., Russell, S.R., Sohn, E.H., et al. (2018). Assessment of adeno-associated virus serotype tropism in human retinal explants. Hum. Gene Ther. *29*, 424–436.

Wright, K.J., Baye, L.M., Olivier-Mason, A., Mukhopadhyay, S., Sang, L., Kwong, M., Wang, W., Pretorius, P.R., Sheffield, V.C., Sengupta, P., et al. (2011). An ARL3-UNC119-RP2 GTPase cycle targets myristoylated NPHP3 to the primary cilium. Genes Dev. *25*, 2347–2360.

Xiao, X., Li, J., and Samulski, R.J. (1998). Production of high-titer recombinant adeno-associated virus vectors in the absence of helper adenovirus. J. Virol. *72*, 2224–2232.

Zhang, H., Constantine, R., Vorobiev, S., Chen, Y., Seetharaman, J., Huang, Y.J., Xiao, R., Montelione, G.T., Gerstner, C.D., Davis, M.W., et al. (2011). UNC119 is required for G protein trafficking in sensory neurons. Nat. Neurosci. *14*, 874–880.

Zhang, H., Hanke-Gogokhia, C., Jiang, L., Li, X., Wang, P., Gerstner, C.D., Frederick, J.M., Yang, Z., and Baehr, W. (2015). Mistrafficking of prenylated proteins causes retinitis pigmentosa 2. FASEB J. *29*, 932–942.

Zhong, X., Gutierrez, C., Xue, T., Hampton, C., Vergara, M.N., Cao, L.H., Peters, A., Park, T.S., Zambidis, E.T., Meyer, J.S., et al. (2014). Generation of three-dimensional retinal tissue with functional photoreceptors from human iPSCs. Nat. Commun. *5*, 4047.

Stem Cell Reports, Volume *15*

Supplemental Information

Modeling and Rescue of RP2 Retinitis Pigmentosa Using iPSC-Derived Retinal Organoids

Amelia Lane, Katarina Jovanovic, Ciara Shortall, Daniele Ottaviani, Anna Brugulat Panes, Nele Schwarz, Rosellina Guarascio, Matthew J. Hayes, Arpad Palfi, Naomi Chadderton, G. Jane Farrar, Alison J. Hardcastle, and Michael E. Cheetham

Supplemental Information

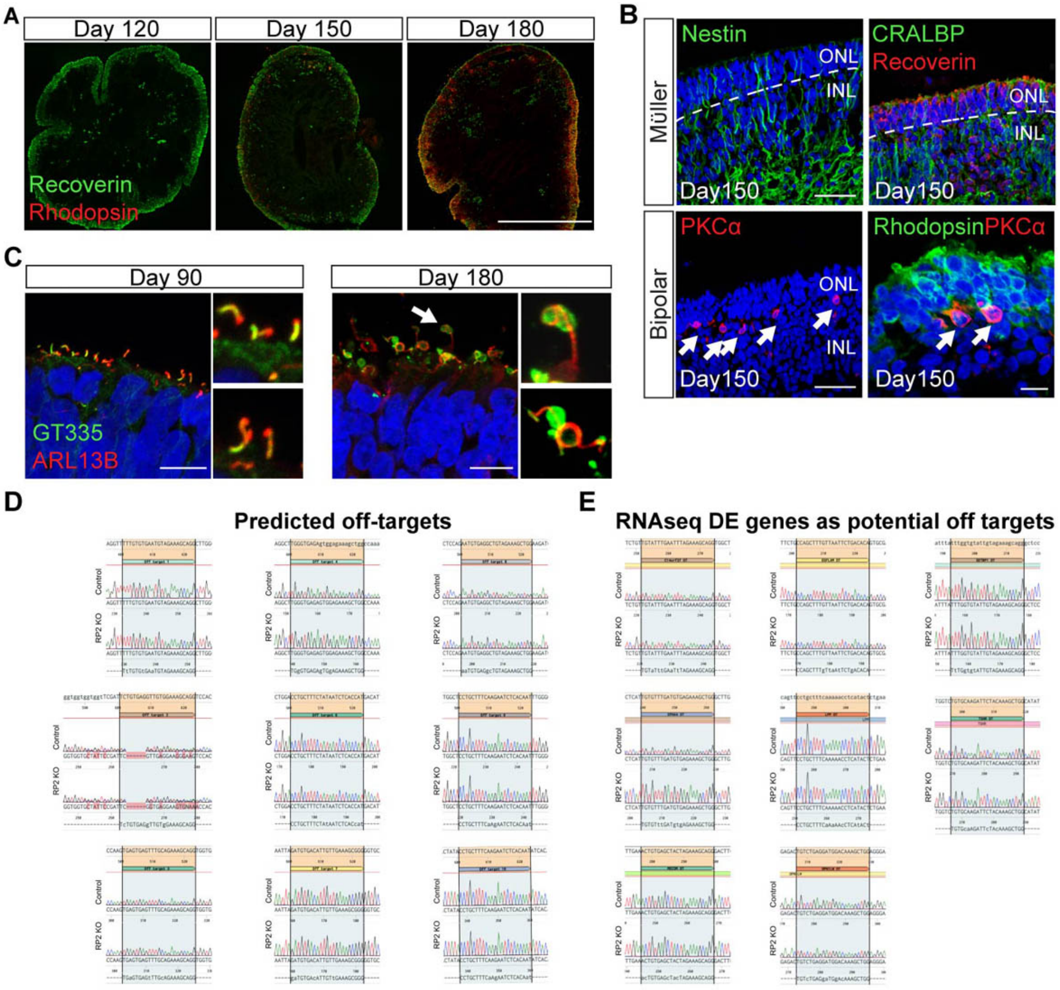

Figure S1. ***Control retinal organoid differentiation and lack of off-target editing in the RP2 KO.*** Related to Figure 1 and experimental methods.

A. ROs show increased rhodopsin expression between D150 and D180 Scale bar =500 µm.

B. ROs contain Muller glia, stained with nestin (green) and CRALBP (green) and bipolar cells, stained with PKCα Scale bars left = 50 µm; lower right = 10 µm**.**

C. Apical cilia in ROs stained with ARL13b (red) and GT335 (green) Scale bars = 10 µm.

D. Sanger sequencing results of Top 10 predicted off-targets for RP2 gRNA2. No mutations were observed in any of the predicted off-target sites. Off target 2 happens to occur in a region where the control line has a naturally occurring variation. Lower case letters in the off-target sequences below the traces indicate the sites at which the off-targets vary from RP2 gRNA2.

E. Sequencing results of RNAseq DE genes with possible similarity to the gRNA sequence. Sanger sequencing revealed that none of these sites showed any editing by off-target Cas9 activity.

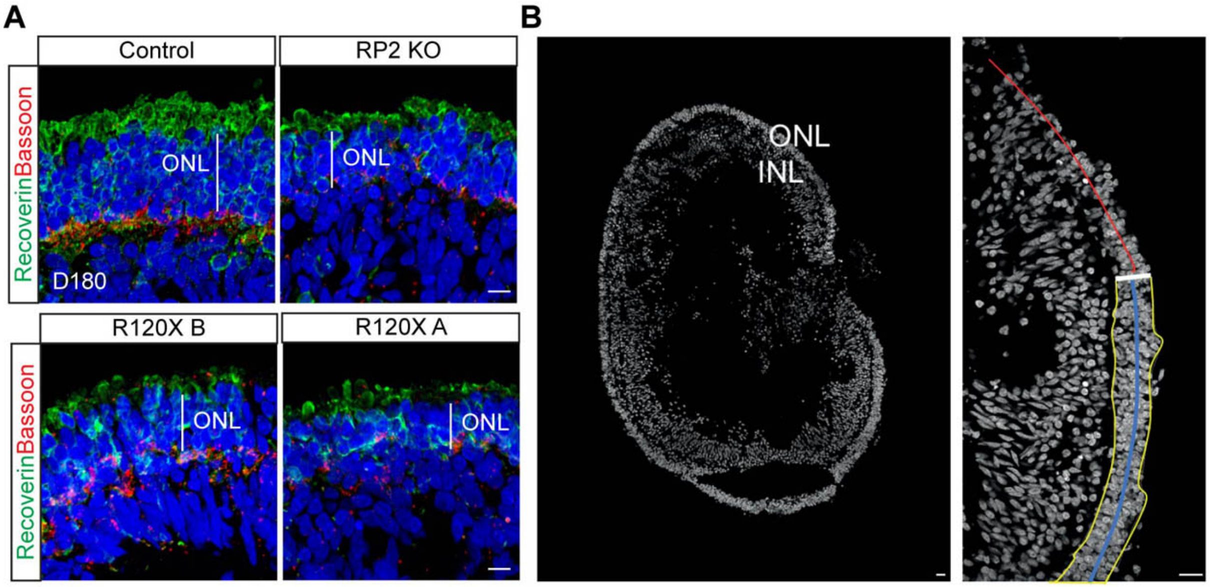

Figure S2. ***Thinning of the ONL in RP2 null cells.*** Related to Figure 2.

A. Representative images showing reduced ONL thickness in RP2 KO, R120X A and R120X B ROs. The Control and RP2 KO images are also shown in Figure 2. Scale bar = 10 μm.

B. Tilescans of retinal organoid to illustrate the method of ONL measurement. Whole organoid (left panel) close up of ONL (right panel). The area of the ONL is enclosed within the yellow marked area and the length of ONL indicated by the blue line. The red line indicates an area of non-photoreceptors that were not included. Scale = 20μm

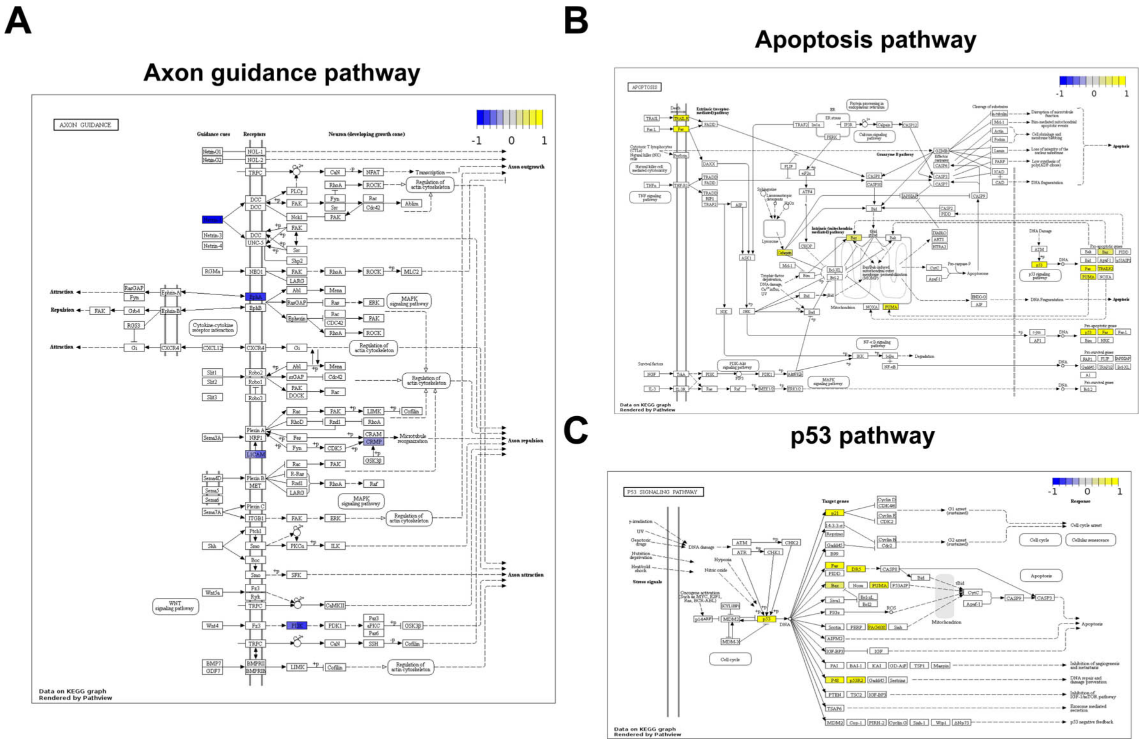

Figure S3. ***KEGG analyses of shared DE genes between RP2 nulls and control.*** Related to Figure 2.

A. KEGG analyses of RP2 KO and RP2 R120X common downregulated DE genes compared to control ROs at D150 in 'axon guidance' pathways.

B. KEGG analyses of RP2 KO and RP2 R120X common DE genes that are upregulated compared to control ROs at D150 in the 'apoptosis' pathway.

C. KEGG analyses of RP2 KO and RP2 R120X common DE genes that are upregulated compared to control ROs at D150 in the 'p53' pathway

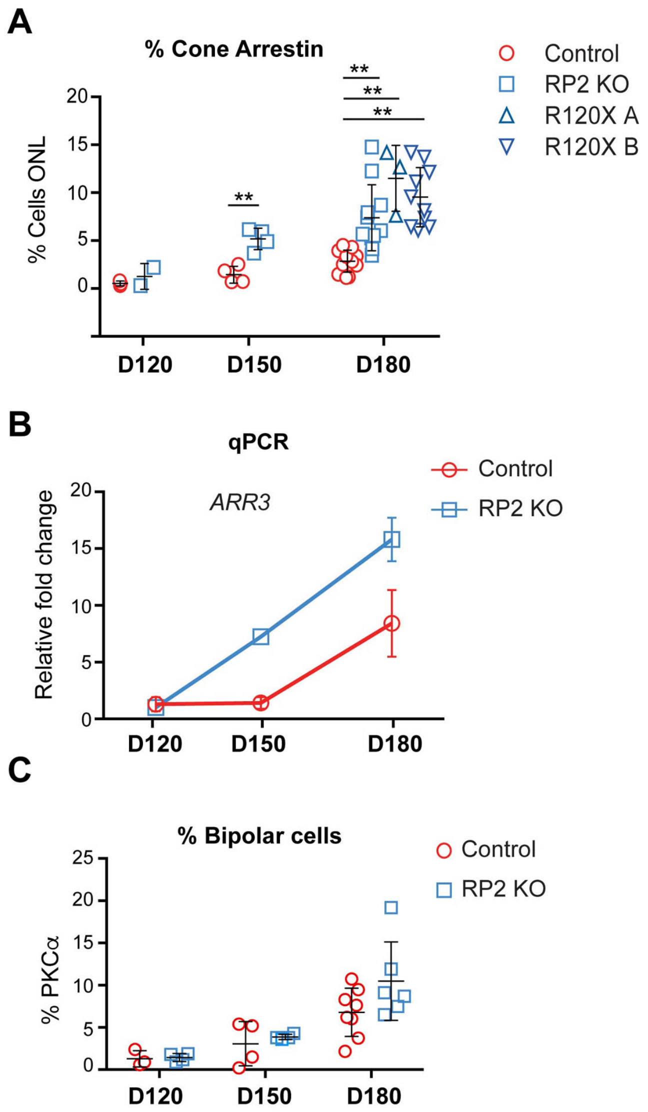

Figure S4. ***Cone photoreceptor and bipolar cells in RP2 null organoids.*** Related to Figure 3.

A. Percent of cells in ONL positive for cone arrestin immunostaining, Control (red circle; n=3, D120; n=4 D150 n=10 D180); RP2 KO (blue square; n=2, D120; n=4 D150 n=10 D180) R120X A (blue triangle; n=3 D180), R120X B (blue inverted triangle; n=10 D180); ** $p<0.01$ each n represents the mean from a full tilescan of an independent organoid. Mean ±SD

B. *ARR3* levels in retinal organoids. qPCR showing relative fold change in mRNA in control and RP2 KO retinal organoids at D120, D150 and D180 (n=3,3,4 independent organoids respectively; mean ±SEM).

C. Cell counts of bipolar cells (positive for PKCα) in control and RP2 KO ROs showed no significant differences in bipolar cell numbers at D120, D150 or D180 (controls n=3, 4 and 8 RP2 KO n=4, 4, 6, mean of tilescan of full independent organoids; mean ±SEM).

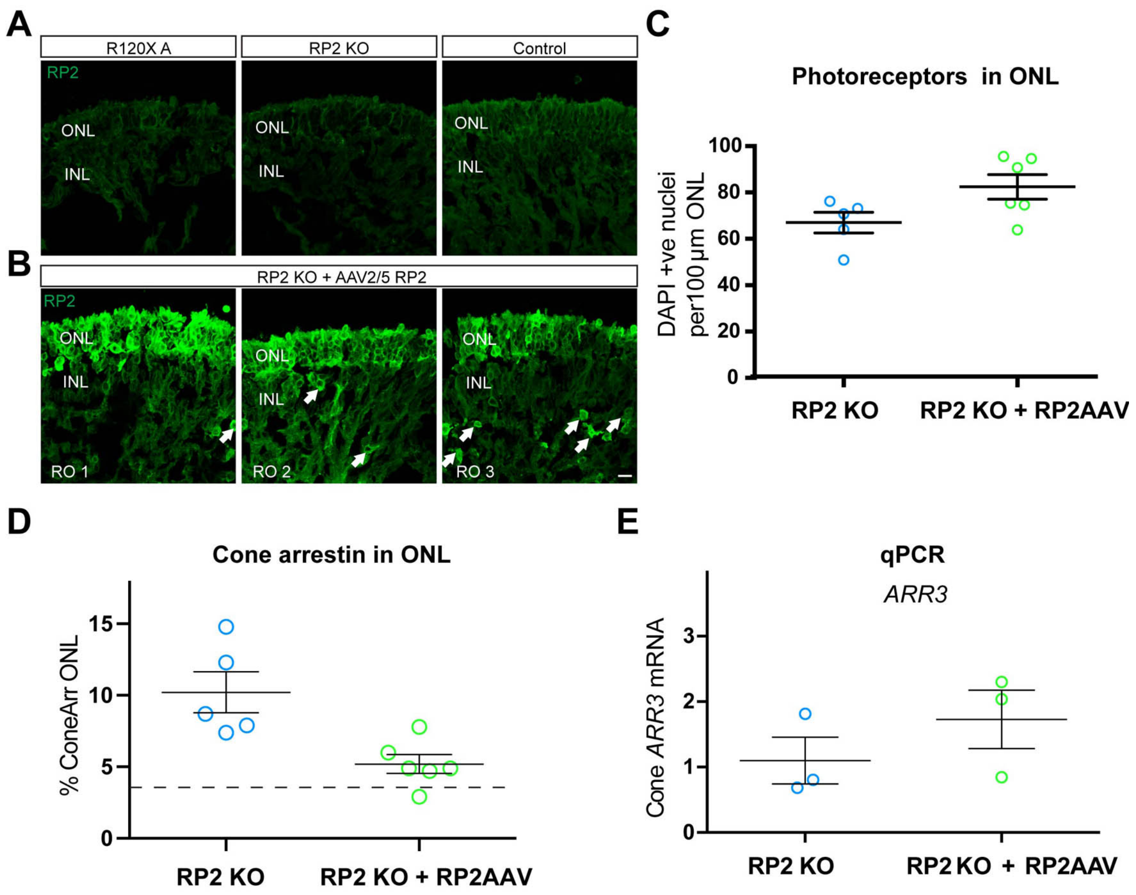

Figure S5. ***Transduction of RP2 KO organoids with RP2-AAV.*** Related to Figure 5.

A. ICC for RP2 in untreated D180 RP2 R120X A and RP2 KO and control ROs.

B. RP2 immunostaining after RP2-AAV transduction in 3 different organoids (RO1, RO2 and RO3). The few strongly positive cells for RP2 outside of the ONL are indicated with arrows. Scale bar = 10 µm.

C. The number of photoreceptors in the ONL. Mean of nuclei in the ONL per 100 µm (n=5 RP2 KO and n=6 RP2 KO + RP2AAV independent organoids for each condition; p=0.058; mean ±SEM).

D. Percentage of cells in the ONL that were positive for cone arrestin staining in untreated and after RP2-AAV transduction at D180, the dotted line indicates the mean percent in controls (RP2 KO n=5, RP2+RP2AAV n=6 independent organoids; p=0.055; mean ±SD).

E. *ARR3* levels in retinal organoids following RP2-AAV transduction. qPCR showing relative fold change in mRNA at D180 (n=3 independent organoids for both conditions; mean ±SEM).

Table S1. gRNA, Primer and Off-target sequences. Related to Figures 1, 4, and S1

Target	Forward	Reverse
**gRNA sequence for RP2 exon 2 CRISPR**		
RP2 gRNA2	TGTGTGAGATTGTAGAAAGC**TGG**	
**PCR primer sequences**		
RP2 exon2	CCAAAAGACTACATGTTCAGTGGA	GCATGATTCATCGCTGCTCT
**Off-target PCR Primer sequences**		
OT1 – Chr13 + 99786425 - 99786447	GGTCAGGGCCAGCACATAAT	TAGCTAATGTGCCCTCAGCG
OT2 - Chr 6 + 38143360 – 38143382 (BTBD9 pre-mRNA)	ACCACGAGGGAGTTAGAGGA	AGCCACATGAGGTTGAAGTCC
OT3 - Chr 9 + 91974085 – 91974107 (SECISBP2 pre- mRNA)	AGTTCTTGCCTGTGTGTCTGT	TAGCAGAACCCCCACTACGC
OT4 - Chr 7 + 157998199 – 157998221 (PTPRN2 pre-mRNA)	TAGCCAAAGCTCTTGCTTCAGT	GTGCCCTATCCTACTTGGCTG
OT5 - Chr 3 – 132906887 -132906909 (TMEM108 pre-mRNA)	CTGTCAAGGAAGGAAGGGAGG	GGAGAAAGGGCCCCCTATAA
OT6 - Chr 5 – 116400519 - 116400541	AACTGTGTCCCACAATAAGGGG	TGCTGGGACCTCCTCAAATTC
OT7 - Chr X + 119452379 - 119452401	AAGCCCACAGGAACTGGGTT	AAACCAAAGTAGGTGGGTCCT
OT8 - Chr 10 + 22037281 - 22037303	ATTCTGCCGGGGTTTAGAGC	AGCAAGGTGAACTGACACTGG
OT9 - Chr 1 – 23887266 - 23887288	AGTAGCTCCAGCTCCATCTGA	ATCTTTCTTGGGCCACAGAGC
OT10 - Chr 11 – 109695393 - 109695415	AAAATGGCTATGCAGCACAAGTT	TTAGCTTCTCCCTTACACCTGAAC
**RNAseq DE gene potential off-target PCR Primer sequences**		
C14orf37	ACTGGGCTTTGAAAGATGGAGA	AGGCACTCAAATGGTGGAGC
EGFLAM	TAGGGCTTTGCACATCGCA	CGGGTTTAGTGGGAAGGCAG
EPHA4	CCTTGTCGATCTTCCACTCCC	ATGACGTGTGGAGCACTGAC
LPP	TCCGACTTTGCCTTCCTTCT	CTCTGTGGCACCTATCACAACT
MECOM	AGCTGCCATTGCTTTTTCTCA	TGCTTAGGAAACACACGCCT
OPN1LW	TGGGTAGTGGTCTTCCCCTC	AGCCCATTTGTGAAGACGGA
SETBP1	TTTCATGCCAGTGACCTTCG	TTGCTCATTGACTTTTGACATGGG
TSHR	AGCCACAACCACTCTTGACT	TGACTGGTTTTCAGGTGCTAGAT

Table S2. Primer sequences for qPCR. Related to Figures 3, 4, 5 and S4, and S5

qPCR primer sequences		
**Target**	**Forward**	**Reverse**
RHO	GGTGGTGTGTAAGCCCATGA	CCTCGGGGATGTACCTGGAC
GNAT1	CTGCTCACTCTGTCCCTTCG	TTGACGATGGTGCTCTTCCC
RP2 EX1-2	GACCATGGGCTGCTTCTTCT	ACTGTTGTCCTGCTACCGTC
ARR3	CATGTGCCTTTCGCTATGGC	TCAATCCCACAGGGCTTTCC
MAN1B1	ACCGTGGAGAGCCTGTTCTA	GTTTGGGTCATCGGAGAAGA
GAPDH	CCCCACCACACTGAATCTCC	GGTACTTTATTGATGGTAC
POLR2A	GCGGGGTGAAGTGATGAAC	ATCATCGGGATGGGTGCTGT

Table S3. List of DE genes from D150 RNAseq analyses. Related to Figure 2.
See excel file Supplementary Table S3.xls.

Table S4. List of antibodies used. Related to all figures.

Marker	Species	Dilution	Supplier	Catalog number
Oct4	Rabbit	1:1000	Abcam	ab19857
Nanog	Mouse	1:1000	Thermo Invitrogen	MA-1-017
RP2 (western blot)	Sheep	1:2500	Sheep serum	References 1, 2
RP2 (ICC)	Rabbit	1:200	ProteinTech	14151-1-AP
Recoverin	Rabbit	1:500	Millipore	AB5585
Cone Arrestin (7G6)	Mouse	1:100	Gift from Wolfgang Baehr	
Rhodopsin (4D2)	Mouse	1:500	Millipore	MABN15
F-actin	AlexaFluor 488 Phalloidin		Thermo Invitrogen	A12379
TOM20	Mouse	1:150	Santa Cruz	SC17764
GT335	Mouse	1:800	Adipogen Life Sciences	AG-20B-0020
Arl13B	Mouse	1:1000	Abcam	ab136648
Bassoon	Mouse	1:500	Enzo	SAP7F407
Nestin	Mouse	1:200	Abcam	ab22035
CRALBP	Mouse	1:300	Thermo Invitrogen	MA1-813
PKCα	Rabbit	1:200	Abcam	ab32376

Supplemental Experimental Procedures

Gene editing off targeting assessment

Possible off-targets for the RP2 gRNA2 were predicted using the Off-Spotter online tool (https://cm.jefferson.edu/Off-Spotter/). Sequences from the RP2 KO line and the isogenic control line were aligned using Benchling (www.benchling.com). No mutations were observed in any of the predicted off-target sites. Off target 2 (Figure S1) happens to occur in a region where the control line has a naturally occurring variation. Primers for Off target 5 failed to amplify the DNA due to low complexity and high GC content in the target region. In addition, the list of DE genes generated by RNAseq analysis of RP2 KO vs control ROs was cross referenced with the list of Off-targets for RP2 gRNA2 (Off-spotter online tool; 431 off-targets predicted). Sanger sequencing revealed that none of these sites showed any editing by off-target Cas9 activity.

AAV production

The following sequences were used in the construct: CMV IE enhancer (661-1024 bp from GenBank: X03922), Ch-B-Actin proximal promoter/intron 1 (251-1542 bp of GenBank: X00182; 1538-39 bp AG was changed to CA); RP2 CDS (190-1270 bp of RefSeq: pNM_006915.2), RP2 3'UTR (1271-2440 bp of RefSeq: NM_006915.2), RP2 poly(A) (50341-50580 bp of RefSeq: NG_009107.1) and minimal rabbit B-globin poly(A) (Levitt et al., 1989)[3].

ONL measurement methods

Mean ONL measurements for control, RP2 KO and R120X patient derived ROs were assessed at three time points (D120, D150 and D180). Each data point represents the mean ONL measurement for an individual unique organoid. Mean ONL measurements were performed on confocal images taken with a 40x objective on a Zeiss LSM710. Organoids varied in size but approximately 12-20 images were acquired with the 40x objective so that the entire organoid section was imaged in a series of tiles. Each tile consisted of a maximum intensity projected Z stack. These tiles were then merged to produce a composite of the entire ONL in each organoid and processed in image J (FIJI). In mature ROs the ONL layer is easily determined by the organisation of nuclei in the DAPI channel, but this was also verified by examining the termination of photoreceptor marker staining (e.g. recoverin, rhodopsin, cone arrestin). Furthermore, between the densely packed nuclei of the ONL there is a physical gap to the INL (Fig. S2), this synaptic outer plexiform layer is labelled with bassoon in Fig 2A and S2. In imageJ (FIJI) the polygon selection tool was used to measure the area around the entire organised ONL in the DAPI channel (yellow line in Fig. S2B). In organoids where the full section was not organised into ONL/INL (e.g if an uncharacterised non-retinal area was present) the junction between retina and non-retina where the ONL tapers away was excluded in all cases (red line in Fig. S2B). In sections where 2 or more retinal 'lobes' were separated by non-retinal tissue, the lobes were measured separately and averaged into one number. The segmented line tool was used to draw a line through the centre of the selection area and the length (in pixels) measured (blue line Fig. S2B). The mean ONL thickness was then determined by division of the total area by the length. In this way deviations in ONL thickness throughout the organoid were accounted for. The final value (in pixels) was then converted to micrometers using for the scaling information from the acquisition format and 40x objective used.

TUNEL assessment method

The percentage of TUNEL positive nuclei in the outer nuclear layer for control, RP2KO and R120X patient derived organoids was derived over three time points, D120, D150 and D180 (Fig. 2D). Each data point represents TUNEL data from an individual unique organoid. TUNEL measurements were carried out on confocal images taken with a 40x objective on a Zeiss LSM710. Organoids varied in size but approximately 12-20 tiles at the 40x objective were acquired so that the entire organoid section was imaged. Each tile consisted of a maximum intensity projected Z stack. These tiles were then merged and opened in image J (FIJI). The area comprising ONL was determined as described above. DAPI nuclei numbers were counted manually in image J. Nuclei were counted in a representative area of ONL for each organoid and the number extrapolated based on total ONL area for each tile scan. TUNEL reactive nuclei within the predetermined ONL area were counted manually in the appropriate channel. The % TUNEL positive nuclei per organoid was then calculated. Across all data points the mean number of nuclei included per organoid was 1522 and the number of TUNEL positive nuclei counted was between 1 and 43.

Supplemental References

1. Chapple JP, Hardcastle AJ, Grayson C, Spackman LA, Willison KR, Cheetham ME (2000) Mutations in the N-terminus of the X-linked retinitis pigmentosa protein RP2 interfere with the normal targeting of the protein to the plasma membrane. Hum Mol Genet 9: 1919-26

2. Grayson C, Bartolini F, Chapple JP, Willison KR, Bhamidipati A, Lewis SA, Luthert PJ, Hardcastle AJ, Cowan NJ, Cheetham ME (2002) Localization in the human retina of the X-linked retinitis pigmentosa protein RP2, its homologue cofactor C and the RP2 interacting protein Arl3. Hum Mol Genet 11: 3065-74
3. Levitt, N., Briggs, D., Gil, A. & Proudfoot, N. J. (1989). Definition of an efficient synthetic poly(A) site. *Genes Dev,* 3**,** 1019-25.
